# TBHP-mediated highly efficient dehydrogenative cross-oxidative coupling of methylarenes with acetanilides

**DOI:** 10.3762/bjoc.12.217

**Published:** 2016-10-25

**Authors:** Cui Chen, Weibing Liu, Peng Zhou

**Affiliations:** 1 College of Chemical Engineering, Guangdong University of Petrochemical Technology, 2 Guandu Road, Maoming 525000, P. R. China. Fax: +86-668-2923575; Tel: +86-668-2923444

**Keywords:** dehydrogenative cross oxidative coupling, methyl arenes, *N*-arylbenzamides, TBHP

## Abstract

A TBHP-mediated dehydrogenative cross-oxidative-coupling approach has been developed for the synthesis of *N*-arylbenzamides from methylarenes and acetanilides. This cross-coupling method is free of transition metal catalysts and ligands, and no extra organic solvents are required, which make it an useful and attractive strategy for the straightforward construction of C–N bonds. Besides, this conversion is an important complement to the conventional C–N forming strategies.

## Introduction

**Scheme 1:**
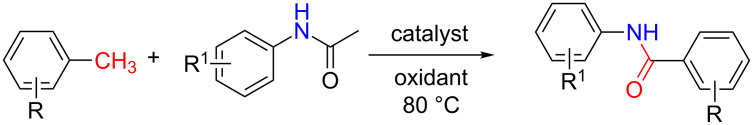
Synthesis of *N*-arylamides.

Recently, amides have attracted more and more attention due to their extensive utilization in pharmaceutical and agrochemical applications [1–3], as well as for precursors in organic synthesis for the construction of natural products, polymers and organic materials [4–6]. The database of medicinal chemistry indicates that around 25% of synthetic drugs contain the amide moiety [7]. What is more, the amide motif has also served as pivotal intermediate to generate several other organic functionalities [8–9]. To date, a large number of amidation reactions have been established [10–11], such as the condensation of carboxylic acid derivatives with amines [12], the rearrangement of ketoximes [13], the C–N cross coupling between aromatic amides or amines and aryl(pseudo)halides [14–15] or aldehydes [16–20]. However, to the best of our knowledge, the studies of dehydrogenative cross-oxidative-coupling reactions between methylarenes and amines for the formation of amides are rather limited, and which would be an important complement to the conventional C–N forming strategies. Herein, we disclose a dehydrogenative C–N cross-oxidative-coupling reaction of methylarenes with acetanilides, using TBHP as an oxidant to afford *N*-arylamides in moderate to good yields (Scheme 1).

## Results and Discussion

We began by studying the reaction of toluene (**1a**) and acetanilide (**2a**) as model substrates to identify the optimal conditions (Table 1). In the presence of I_2_ (0.1 equiv) and *tert*-butyl hydroperoxide (TBHP, 2.0 equiv), the study commenced to optimize the reaction time (Table 1, entries 1–3). The results show that the reaction was completed after 24 h and led to the desired *N*-phenylbenzamide **3aa** in 62% GC yield (Table 1, entry 2). Disappointingly, other peroxides like di-*tert*-butylperoxide (DTBP), benzoyl peroxide, dicumyl peroxide (DCP), methyl ethyl ketone peroxide (MEKP), *tert-*butylperoxy benzoate (TBPB) and cumene hydroperoxide (CHP) proved wholly ineffective for this conversion and no product was observed (Table 1, entries 4–9). Next, the effect of other iodine sources on the reaction was monitored. Pleasingly, ICl and NIS afforded the desired product **3aa** successfully, but led to a marked decrease in yield (Table 1, entries 10 and 11). When the loading of I_2_ was increased to 1.0 equivalent, a pronounced improvement in the reaction yield was observed (Table 1, entry 12). Further increasing the loading of I_2_ did not show any beneficial effect (Table 1, entry 13). However, the reaction does not occur in the absence of molecular iodine (Table 1, entry 14), which indicates that molecular iodine is requisite for this conversion. We were pleased to find that an excellent product yield of 86% was obtained (Table 1, entry 15) when increasing the loading of TBHP to 3.0 equivalents. However, decreasing the loading of TBHP to 1.0 equivalent led to a drastic drop in the yield (Table 1, entry 16). It is worth mentioning that this conversion did not show any beneficial effect with nitrogen protection.

**Table 1:** Optimization studies^a^.

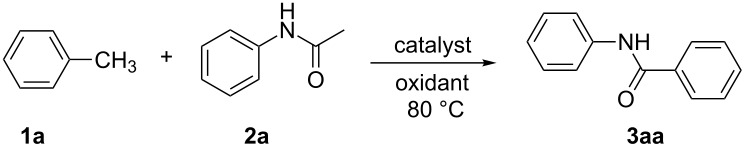				

Entry	Catalyst (0.1 equiv)	Oxidant (2.0 equiv)	Time (h)	Yield (%)^b^

1	I_2_	TBHP	12	47
2	I_2_	TBHP	24	62
3	I_2_	TBHP	36	62
4	I_2_	DTBP	24	0
5	I_2_	benzoyl peroxide	24	0
6	I_2_	DCP	24	0
7	I_2_	MEKP	24	0
8	I_2_	TBPB	24	0
9	I_2_	CHP	24	0
10	ICl	TBHP	24	31
11	NIS	TBHP	24	39
12^c^	I_2_	TBHP	24	71
13^d^	I_2_	TBHP	24	71
14	–	TBHP	24	–
15^c,e^	I_2_	TBHP	24	86
16^c,f^	I_2_	TBHP	24	37

^a^Unless otherwise specified, all the reactions were carried out on **2a** 0.25 mmol scale, catalyst 0.1 equivalents, oxidant 2.0 equivalents, toluene 2.0 mL; ^b^GC yield; ^c^iodine 1.0 equivalents; ^d^iodine 1.5 equivalents; ^e^TBHP 3.0 equivalents; ^f^TBHP 1.0 equivalent.

After identifying the optimized conditions, we next explored the substrate scope of this transformation. As detailed in Table 2, a wide variety of acetanilides having substituent groups such as methyl, methoxy, ethoxyl, chloro and cyano at different positions were employed to react under the standard conditions. We were pleased to find that all these tested substrates were successfully converted into the desired *N*-arylbenzamides **3**. Notably, this conversion appears quite sensitive with respect to the nature (electron-donating or electron-withdrawing) and positions of the substituent groups under the stipulated conditions. *para*-Methoxy- and *para*-ethoxy-substituted acetanilides led to a drastic drop in yield as compared to *para*-cyano- and *para*-chloro-substituted acetanilides (Table 2, entries 5–8). As well as *ortho*-methyl- and *ortho*-chloro-substituted substrates also led to a marked decrease in yield as compared to their corresponding *para*-substituted substrates (Table 2, entries 2, 3, 5, and 9). To further extend the adaptability of this transformation, other methyl arenes were also checked under the standard conditions. Pleasingly, *m*-xylene and *p*-xylene afforded the desired product **3** successfully, but in lower yields (Table 2, entries 10 and 11). In the same manner, we next investigated the reactions of *p*-xylene with *N*-phenylacetamide, *N*-*p*-tolylacetamide, *N*-(4-methoxyphenyl)acetamide, *N*-(2-chlorophenyl)acetamide and *N*-(3-chlorophenyl)acetamide under the standard conditions. Gratifyingly, all the tested acetamides reacted with *p*-xylene successfully, offering the desired *N*-arylbenzamides in moderate yields, from 51% to 69% (Table 2, entries 12–14). It is noteworthy that the reactions did not result in the desired product when using aniline and diethylamine as the partners of acetanilide.

**Table 2:** Scope of the *N*-arylamides^a^.

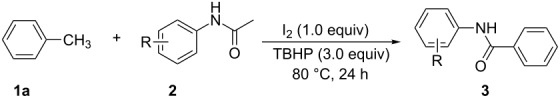			

Entry	**2**	**3**	Yield(%)^b^

1	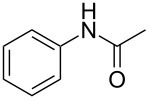	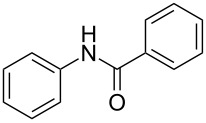	80
2	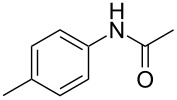	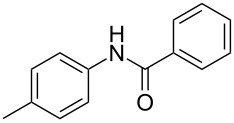	75
3	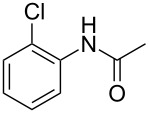	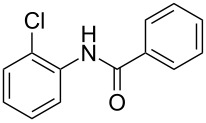	72
4	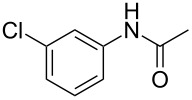	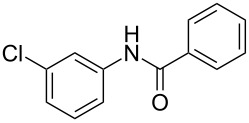	81
5	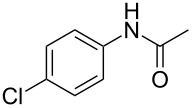	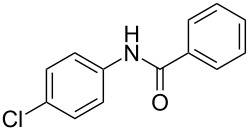	83
6	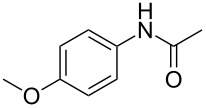	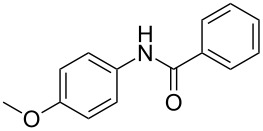	63
7	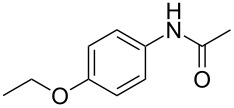	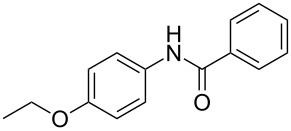	62
8	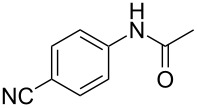	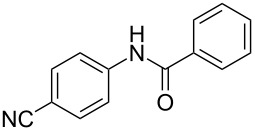	85
9	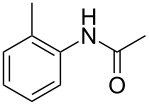	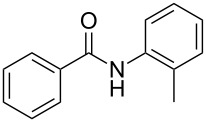	71
10	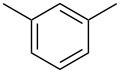	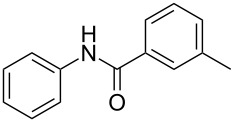	59
11	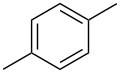	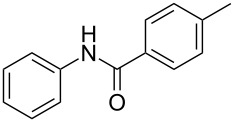	66
12	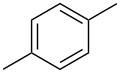	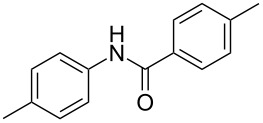	65
13	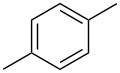	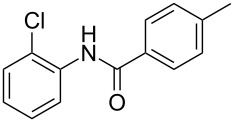	57
14	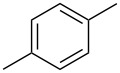	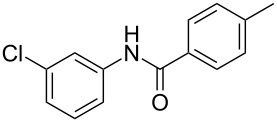	69
15	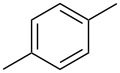	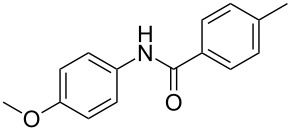	51

^a^Unless otherwise specified, all the reactions were carried out on **2** 1.0 mmol scale, **1** 2.0 mL; ^b^isolated yield.

**Scheme 2:**
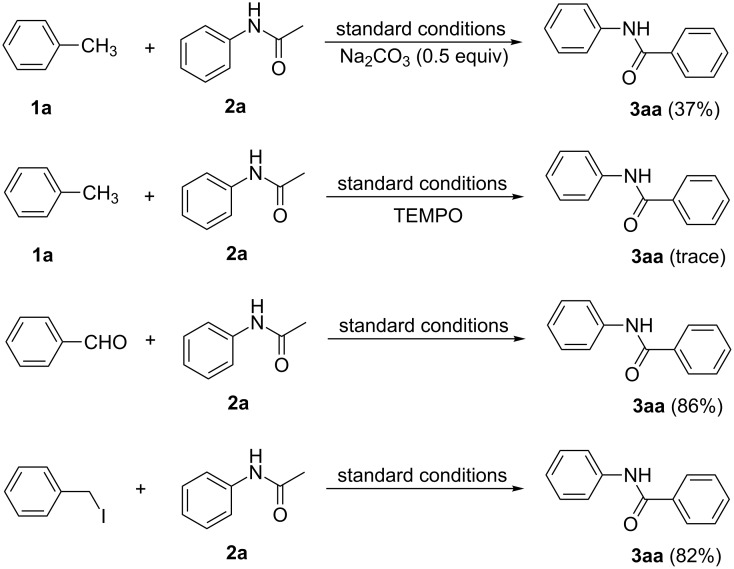
Control experiments.

In order to gain insight into the nature of this conversion, two experiments were conducted (Scheme 2). With the addition of Na_2_CO_3_ (sodium carbonate) into the reaction between **1a** and **2a**, the yield of **3aa** decreased dramatically to 37%, which confirmed that Na_2_CO_3_ has an inhibitory effect for this transformation. At the same time, the radical scavenger TEMPO (2,2,6,6-tetramethylpiperidinoxyl) completely inhibited the reaction and almost no product was obtained. The result indicated that the mechanism may involve a radical pathway. To our delight, reactions using benzaldehyde and 1-(iodomethyl)benzene as the surrogates of toluene afforded **3aa** in excellent yields. These results evidence that either 1-(iodomethyl)benzene or benzaldehyde may be the reaction intermediates derived in situ from toluene.

**Scheme 3:**
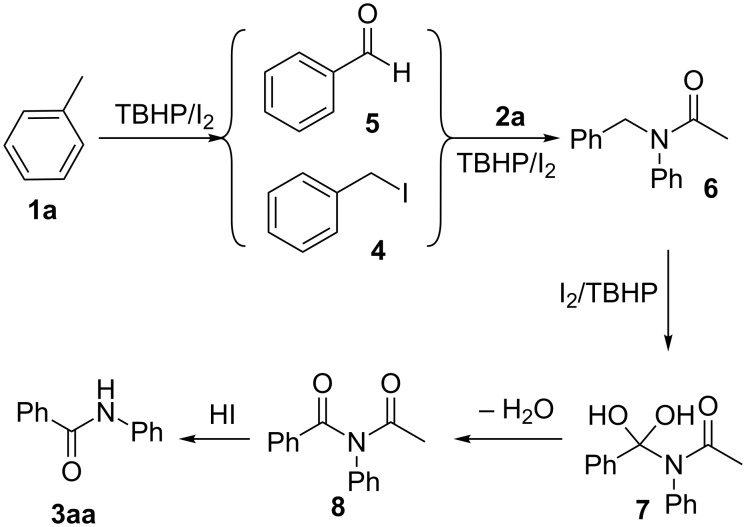
Plausible mechanism.

The mechanism for this conversion is unclear. Based on literature reports and our present experimental results, a plausible reaction mechanism has been proposed in Scheme 3 and exemplified by the formation of **3aa**. Initially, toluene (**1a**) reacted with molecular iodine and TBHP to produce the 1-(iodomethyl)benzene (**4**) and benzaldehyde (**5**) [21–22]. Intermediate **6** was generated from the coupling of **2a** with intermediate **4** [22], by eliminating a molecule of HI. According to the results of the control experiments, intermediate **6** also could be obtained from the reaction of benzaldehyde with **2a** under the standard conditions. Then, intermediate **6** underwent a sequence of steps including fast oxidation and dehydration to give *N*-acetyl-*N*-phenylbenzamide (**8**) under the stipulated conditions**,** similar to the report of An [23]. Finally, the acidic hydrolysis of *N*-acetyl-*N*-phenylbenzamide (**8**) furnished the final product *N*-phenylbenzamide (**3aa**).

## Conclusion

In conclusion, a dehydrogenative C–N cross oxidative coupling approach has been developed for the synthesis of *N*-arylbenzamides from methylarenes and acetanilides. In this protocol, the C–N cross oxidative coupling is free of transition metal catalysts, which makes the present method a useful and attractive strategy for the straightforward construction of C–N bonds. Besides, this conversion is an important complement to the conventional C–N forming strategies. More applications of this novel protocol and the study of the detailed mechanism are currently underway.

## Supporting Information

File 1 Full experimental details and copies of NMR spectral data.
