# Supplementary material for: TBHP-mediated highly efficient dehydrogenative cross-oxidative coupling of methylarenes with acetanilides
Source: Beilstein J Org Chem. 2016 Oct 25;12:2250–5. doi: 10.3762/bjoc.12.217 (PMC5238673; doi:10.3762/bjoc.12.217)

Supporting Information

for

TBHP-mediated highly efficient dehydrogenative cross-oxidative coupling of methylarenes with acetanilides

Cui Chen*, Weibing Liu and Peng Zhou

Address: College of Chemical Engineering, Guangdong University of Petrochemical Technology, 2 Guandu Road, Maoming 525000, P. R. China. Fax: +86-668-2923575; Tel: +86-668-2923444

Email: Cui Chen* - cc161002@gdupt.edu.cn.

*Corresponding author

Full experimental details and copies of NMR spectral data

General methods. All the reactions were carried out at 80 °C for 24 h in a round-bottom flask equipped with magnetic stir bar. Solvents and reagents were purchased from Aldrich Chemicals or J & K Scientific Ltd, were used as received. Petroleum ether (PE) refers to the fraction boiling in the 60–90 °C range. Thin-layer chromatography was performed using Qingdao-Haiyang 600 mesh silica gel plates (GF254), and samples were made visual with short-wavelength UV light (254 nm). ^{1}H NMR and ^{13}C NMR spectra were recorded on a 400 MHz spectrometer in solutions of $CDCl_3$ using tetramethylsilane as the internal standard, δ values are given in ppm and coupling constants (*J*) in Hz.

Typical procedure: *N*-phenylbenzamide (Table 2, entry 1). A mixture of *N*-phenylacetamide (**2a**, 135 mg, 1.0 mmol), iodine (254 mg, 1 mmol), TBHP (387 mg, 3.0 mmol, 70% in water) and toluene (2.0 mL) was added successively in a round-bottom flask, and the resulting soln. stirred for 24 h at 80 °C. The mixture was then subjected to purification by preparative thin-layer chromatography (PE–EtOAc, 10:3) to afford product **3aa**.

Characterization data for all prepared compounds.

***N*-phenylbenzamide (Table 2, entry 1)[1]**

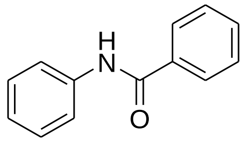

^{1}H NMR ($CDCl_3$, 400 MHz) δ: 7.87 (m, 3H), 7.65 (d, *J*=8.0 Hz, 2H), 7.55 (t, *J*=7.6 Hz, 1H), 7.48 (t, *J*=7.6 Hz, 2H), 7.37 (t, *J*=7.6 Hz, 2H), 7.16 (t, *J*=7.6 Hz, 1H); ^{13}C NMR ($CDCl_3$, 100 MHz) δ: 165.7, 137.9, 134.9, 131.8, 129.1, 128.8, 127.0, 124.5, 120.2.

***N*-(*p*-Tolyl)benzamide (Table 2, entry 2)[2]**

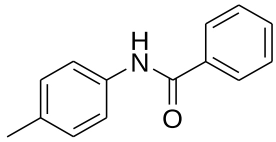

^{1}H NMR ($CDCl_3$, 400 MHz) δ: 7.87 (d, *J*=7.2 Hz, 2H), 7.81 (s, 1H), 7.52 (m, 5H), 7.18 (d, *J*= 8.0 Hz, 2H), 2.35 (s, 3H); ^{13}C NMR ($CDCl_3$, 100 MHz) δ: 165.2, 145.2, 135.7, 134.2, 131.7, 129.5, 128.7, 126.9, 120.3, 20.9.

***N*-(2-Chlorophenyl)benzamide (Table 2, entry 3)[3]**

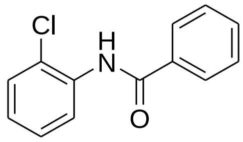

^{1}H NMR ($CDCl_3$, 400 MHz) δ: 8.58 (d, *J*=8.0 Hz, 1H), 8.46 (s, 1H), 7.93 (d, *J*=8.0 Hz, 1H), 7.59(t, *J*=7.6 Hz, 1H), 7.53 (t, *J*=8.0 Hz, 2H), 7.42 (d, *J*=8.0 Hz, 1H), 7.34 (t, *J*=8.0 Hz, 1H), 7.09 (d, *J*=8.0 Hz, 1H); ^{13}C NMR ($CDCl_3$, 100 MHz) δ: 165.2, 134.7, 134.6, 132.2, 129.0, 128.9, 127.9, 127.1, 124.7, 123.0, 121.5.

***N*-(3-Chlorophenyl)benzamide (Table 2, entry 4)[3]**

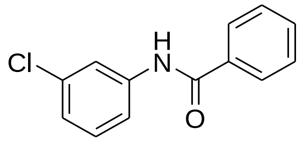

^{1}H NMR ($CDCl_3$, 400 MHz) δ: 8.08 (s, 1H), 7.85 (d, *J*=8.0 Hz, 2H), 7.77 (s, 1H), 7.51 (m, 4H), 7.26 (t, *J*=7.6 Hz, 1H), 7.12 (t, *J*=8.0 Hz, 1H); ^{13}C NMR ($CDCl_3$, 100 MHz) δ: 165.9, 139.1, 134.7, 134.5, 132.0, 130.0, 128.8, 127.0, 124.6, 120.4, 118.2.

N-(4-Chlorophenyl)benzamide (Table 2, entry 5)[1]

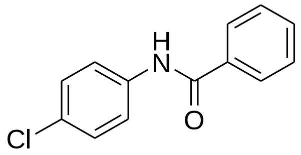

^{1}H NMR ($CDCl_3$, 400 MHz) δ: 8.10 (d, J=8.0 Hz, 2H), 7.90 (s, 1H), 7.65 (t, J=7.6 Hz, 1H), 7.51 (m, 4H), 7.28 (d, J=8.0 Hz, 2H); ^{13}C NMR ($CDCl_3$, 100 MHz) δ: 165.2, 135.2, 133.9, 130.1, 129.8, 129.1, 129.0, 128.8, 128.7, 121.5.

N-(4-Methoxyphenyl)benzamide (Table 2, entry 6)[4]

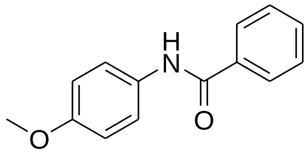

^{1}H NMR ($CDCl_3$, 400 MHz) δ: 7.86 (d, J=8.0 Hz, 2H), 7.73 (s, 1H), 7.53 (d, J=8.0 Hz, 3H), 7.48 (t, J=7.6 Hz, 2H), 6.91 (d, J=8.4 Hz, 2H), 3.82 (s, 3H); ^{13}C NMR ($CDCl_3$, 100 MHz) δ: 164.8, 156.6, 151.8, 131.6, 130.9, 128.7, 126.9, 122.0, 114.2, 55.4.

N-(4-Ethoxyphenyl)benzamide (Table 2, entry 7)[5]

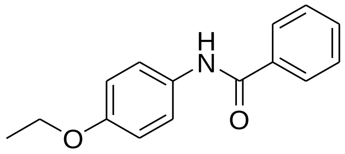

^{1}H NMR ($CDCl_3$, 400 MHz) δ: 7.87 (d, J=8.0 Hz, 2H), 7.74 (s, 1H), 7.50 (m, 5H), 6.90 (d, J= 8.4 Hz, 2H), 4.04 (q, J=7.6 Hz, 2H), 1.42 (t, J=7.6 Hz, 3H); ^{13}C NMR ($CDCl_3$, 100 MHz) δ: 168.6, 155.8, 131.5, 130.5, 128.7, 126.9, 121.9, 114.9, 63.9, 14.8.

N-(4-Cyanophenyl)benzamide (Table 2, entry 8)[6]

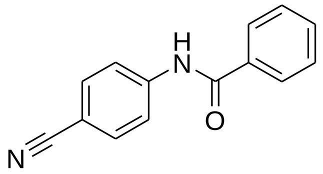

^{1}H NMR ($CDCl_3$, 400 MHz) δ: 8.08 (s, 1H), 7.87 (d, J=8.0 Hz, 2H), 7.80 (d, J=8.0 Hz, 2H), 7.65 (d, J=7.6 Hz, 2H), 7.65 (t, J=7.6 Hz, 1H), 7.51 (t, J=7.2 Hz, 2H); ^{13}C NMR ($CDCl_3$, 100 MHz) δ: 165.8, 142.0, 134.0, 133.3, 132.5, 128.9, 127.1, 119.9, 118.8, 107.3.

N-(o-Tolyl)benzamide (Table 2, entry 9) [7]

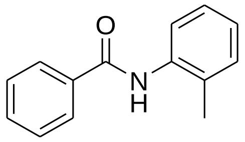

^{1}H NMR ($CDCl_3$, 400 MHz) δ: 7.89 (d, J=7.6 Hz, 2H), 7.77 (s, 1H), 7.57 (t, J=7.6 Hz, 1H), 7.49 (d, J=7.6 Hz, 2H), 7.25 (m, 2H), 7.13 (t, J=7.6 Hz, 1H), 2.33 (s, 3H); ^{13}C NMR ($CDCl_3$, 100 MHz) δ: 165.7, 135.7, 135.0, 131.8, 130.5, 129.4, 128.8, 127.0, 126.8, 125.4, 123.3, 17.8.

N-(m-Tolyl)benzamide (Table 2, entry 10) [7]

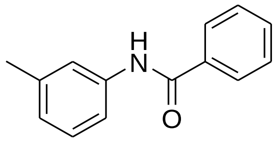

^{1}H NMR ($CDCl_3$, 400 MHz) δ: 7.79 (s, 1H), 7.70 (s, 1H), 7.65 (m, 3H), 7.37 (m, 4H), 7.16 (t, J= 8.0 Hz, 1H), 2.45 (s, 3H); ^{13}C NMR ($CDCl_3$, 100 MHz) δ: 165.9, 145.0, 138.7, 134.3, 132.6, 129.1, 128.6, 127.7, 124.5, 123.9, 120.1, 21.4.

4-Methyl-N-phenylbenzamide (Table 2, entry 11)[1]

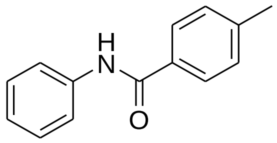

^{1}H NMR ($CDCl_3$, 400 MHz) δ: 7.76 (s, 1H), 7.52 (d, J=7.6 Hz, 2H), 7.32 (t, J=7.6 Hz, 2H), 7.25 (t, J=7.6 Hz, 1H), 7.14 (d, J=7.6 Hz, 2H), 7.09 (d, J=8.0 Hz, 2H), 2.31 (s, 3H); ^{13}C NMR ($CDCl_3$, 100 MHz) δ: 172.8, 143.0, 139.2, 129.5, 129.3, 129.0, 128.4, 127.9, 120.2, 21.5.

4-Methyl-N-(p-tolyl)benzamide (Table 2, entry 12)[7]

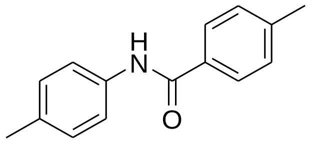

^{1}H NMR ($CDCl_3$, 400 MHz) δ: 7.90 (s, 1H), 7.77 (d, J=7.6 Hz, 2H), 7.53 (d, J=8.0 Hz, 2H), 7.27 (d, J=8.0 Hz, 2H), 7.17 (d, J=7.6 Hz, 2H), 2.43 (s, 3H), 2.35 (s, 3H); ^{13}C NMR ($CDCl_3$, 100 MHz) δ: 165.6, 142.1, 135.5, 134.0, 132.1, 129.5, 129.3, 127.0, 120.3, 21.4, 20.8.

***N*-(2-Chlorophenyl)-4-methylbenzamide (Table 2, entry 13)**[8]

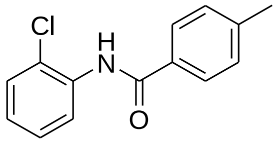

^{1}H NMR ($CDCl_3$, 400 MHz) δ: 7.93 (s, 1H), 7.45 (d, *J*=7.6 Hz, 2H), 7.36 (d, *J*=7.2 Hz, 1H), 7.17 (m, 3H), 7.09 (d, *J*=7.2 Hz, 1H), 7.03 (d, *J*=7.6 Hz, 2H), 2.24 (s, 3H); ^{13}C NMR ($CDCl_3$, 100 MHz) δ: 172.0, 142.8, 137.2, 132.8, 131.9, 130.5, 129.9, 129.1, 128.9, 128.8, 127.8, 21.5.

***N*-(3-Chlorophenyl)-4-methylbenzamide (Table 2, entry 14)**[9]

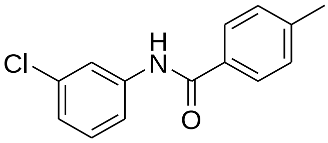

^{1}H NMR ($CDCl_3$, 400 MHz) δ: 8.08 (s, 1H), 7.76 (m, 3H), 7.50 (d, *J*=8.0 Hz, 1H), 7.26 (m, 3H), 7.11 (t, *J*=7.2 Hz, 2H), 2.42 (s, 3H); ^{13}C NMR ($CDCl_3$, 100 MHz) δ: 165.8, 142.6, 139.2, 134.6, 131.6, 129.8, 129.4, 127.1, 124.4, 120.3, 118.2, 21.5.

***N*-(4-Methoxyphenyl)-4-methylbenzamide (Table 2, entry 15)**[7]

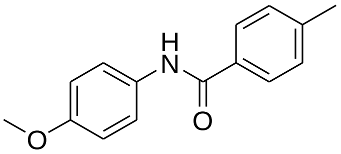

^{1}H NMR ($CDCl_3$, 400 MHz) δ: 7.68 (m, 3H), 7.45 (d, *J*=8.0 Hz, 2H), 7.19 (d, *J*=8.0 Hz, 2H), 6.82 (d, *J*=7.6 Hz, 2H), 2.41 (s, 3H), 2.33 (s, 3H); ^{13}C NMR ($CDCl_3$, 100 MHz) δ: 179.9, 165.4, 142.2, 135.4, 134.0, 132.2, 129.5, 129.4, 127.0, 120.2, 21.4, 20.9 .

References

[1] Roy, S.; Sarma, M. J.; Kashyap, B.; Phukan, P. *Chem. Commun.* **2016**, *52*, 1170-1173.

[2] Iranpoor, N.; Panahi, F.; Roozbin, F.; Erfan, S.; Rahimi, S. *Eur. J. Org. Chem.* **2016**, *9*, 1781-1787.

[3] Tan, B.; Teo, Y. C. *Org. Bio. Chem.* **2014**, *12*, 7478-7481.

[4] Hong, M.; Tang, X. Y.; Falivene, L.; Caporaso, L.; Cavallo, L.; Chen, E. *J. Am. Chem. Soc.* **2016**, *138*, 2021-2035.

[5] Ji, Y. F.; Yan, H.; Jiang, Q. B. *Eur. J. Org. Chem.* **2015**, *9*, 2051−2060.

[6] Dutta, U.; Lupton, D. W.; Maiti, D. *Org. Lett.* **2016**, *18*, 860−863.

[7] Sharma, N.; Sekar, G. *Adv. Synth. Cat.* **2016**, *358*, 314-320.

[8] Demmer, C. S.; Hansen, J. C.; Kehler, J.; Bunch, L. *Adv. Synth. Cat.* **2014**, *356*, 1047-1055.

[9] Lee, B. C.; Yoon, J. H.; Lee, C. G.; Lee, I. *J. Phys. Org. Chem.* **1994**, *7*, 273-279.

NMR spectra

***N*-Phenylbenzamide (Table 2, entry 1)**

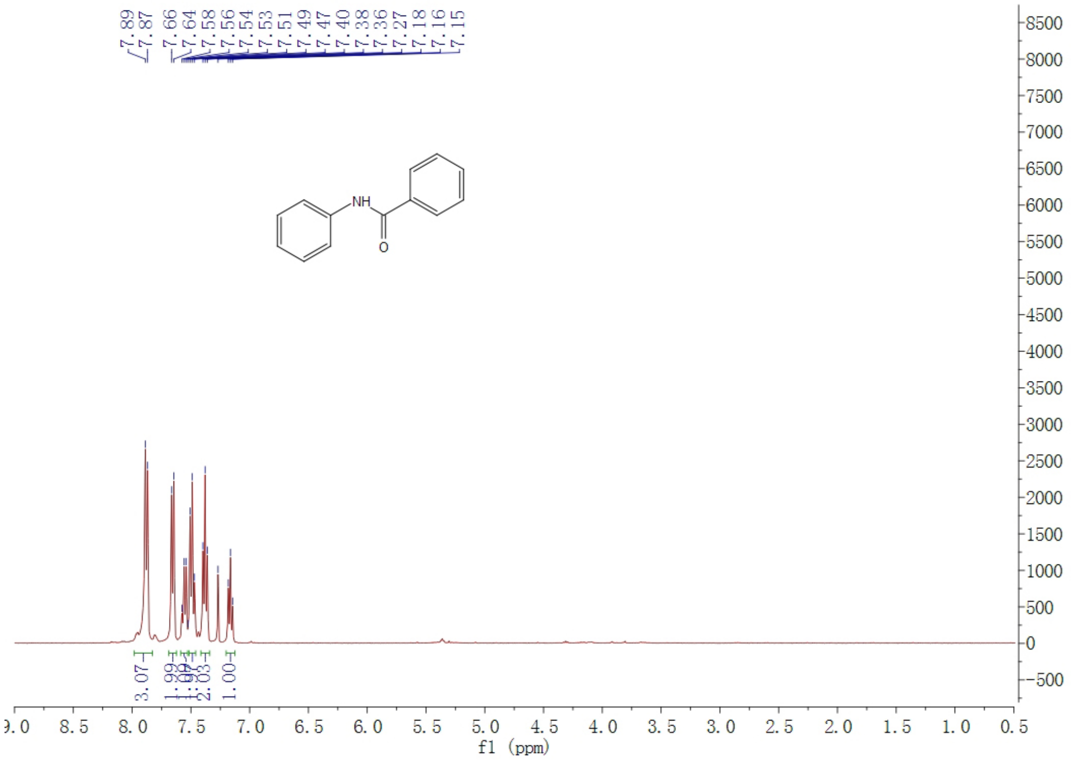

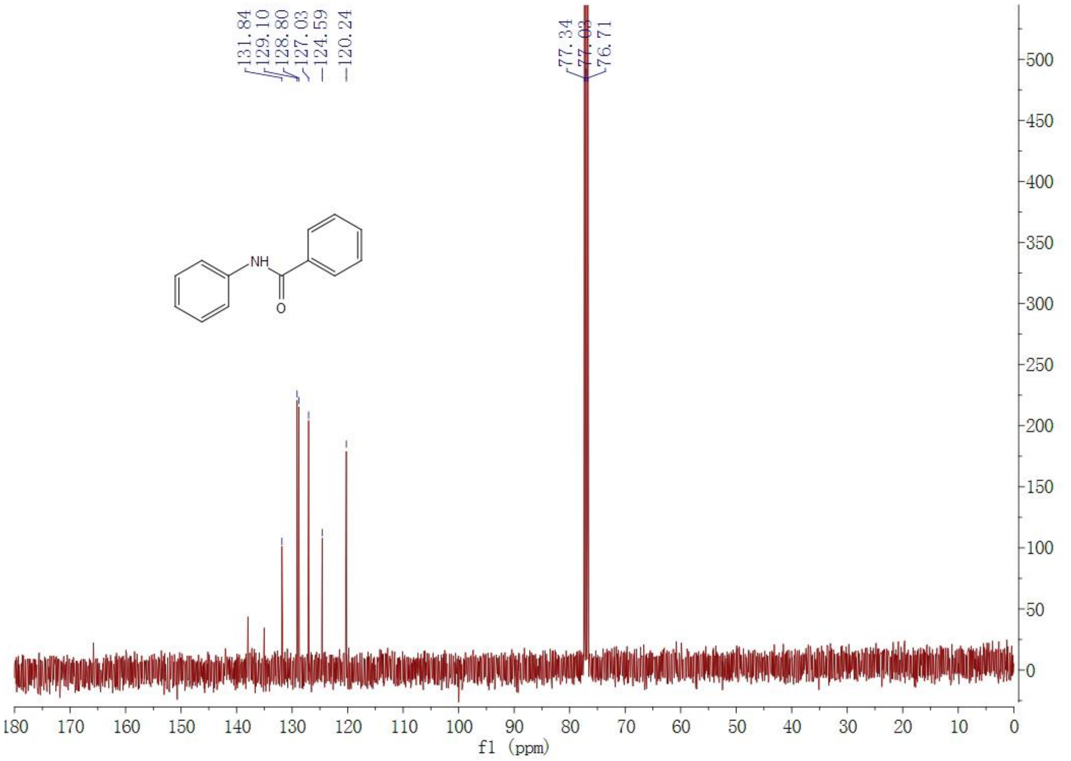

***N*-(*p*-Tolyl)benzamide (Table 2, entry 2)**

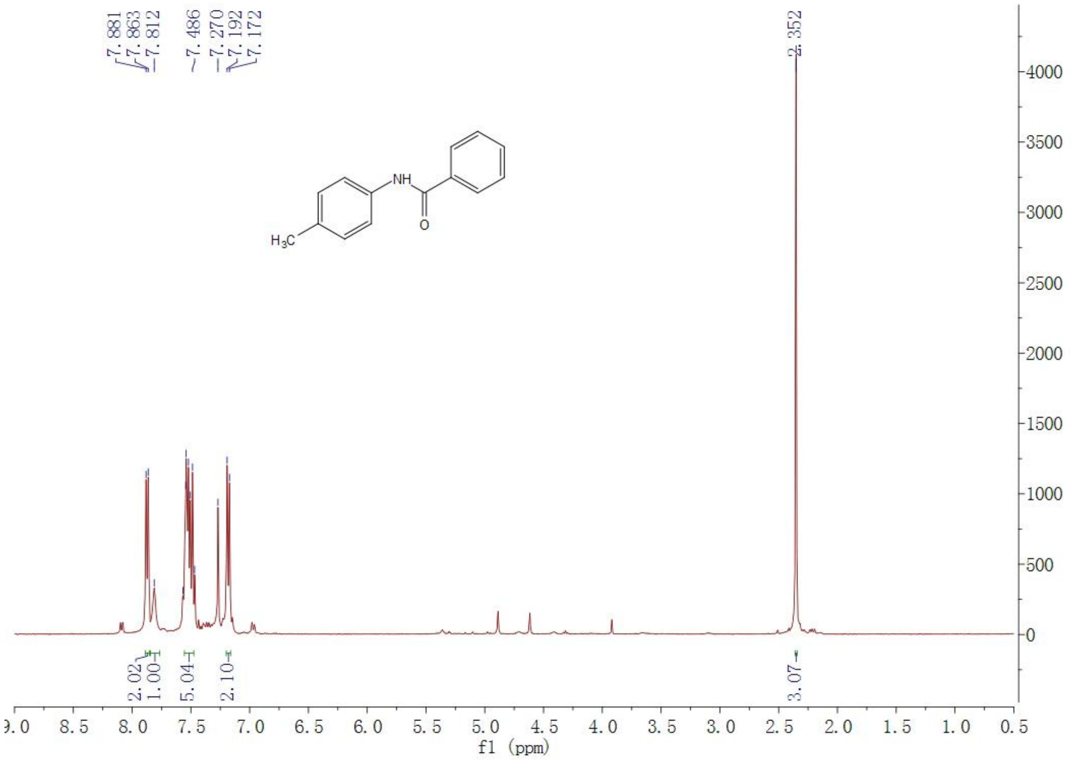

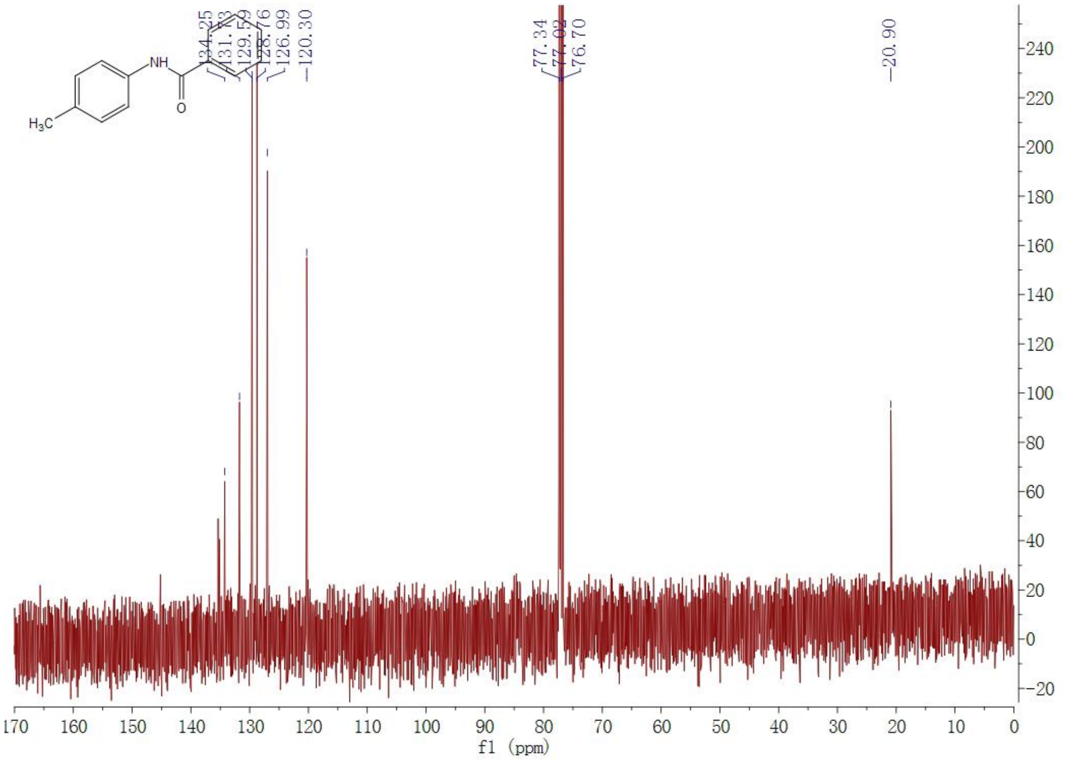

N-(2-Chlorophenyl)benzamide (Table 2, entry 3)

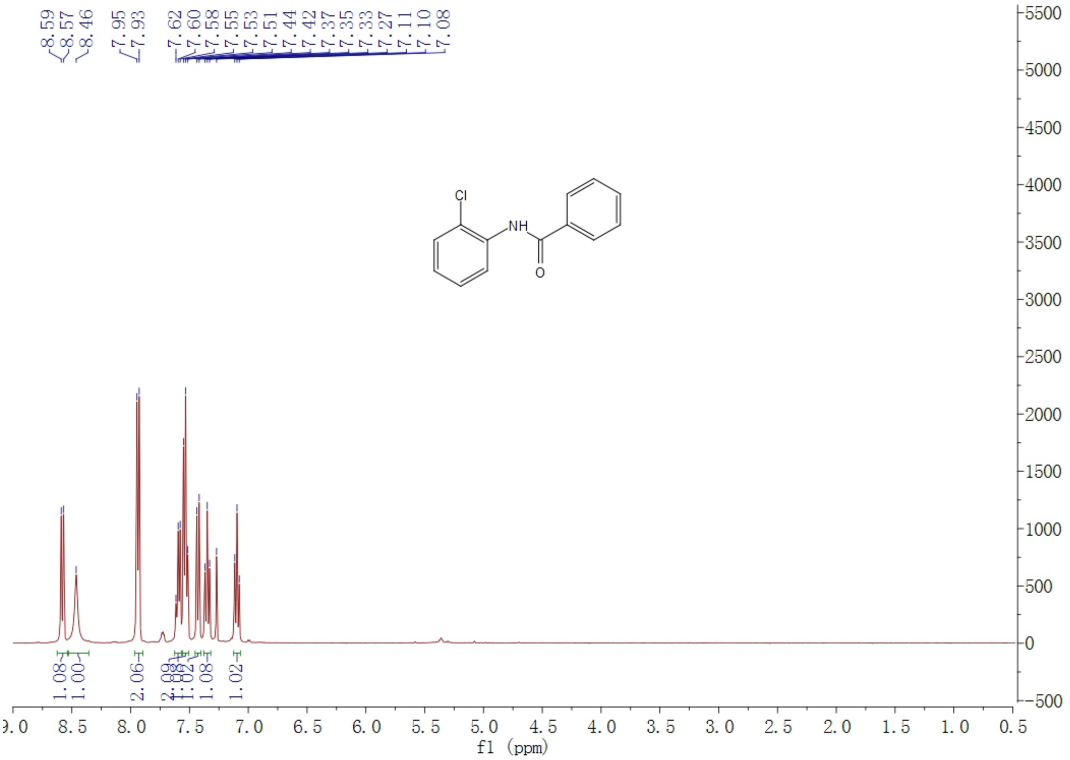

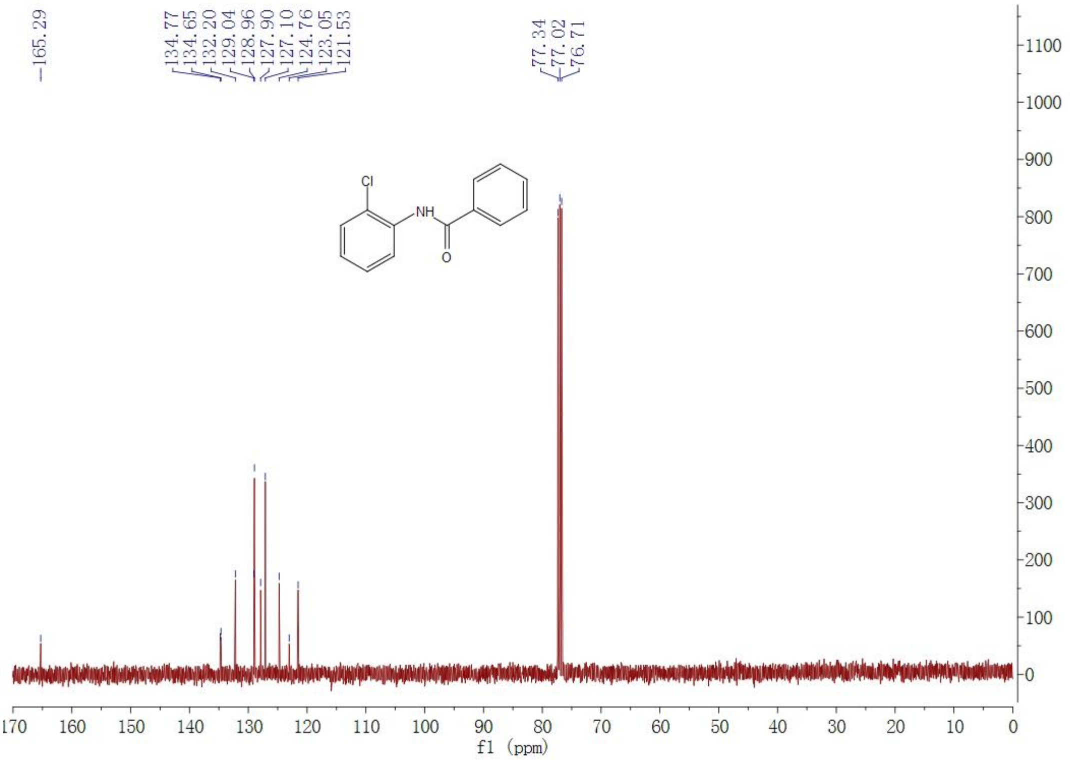

***N*-(3-Chlorophenyl)benzamide (Table 2, entry 4)**

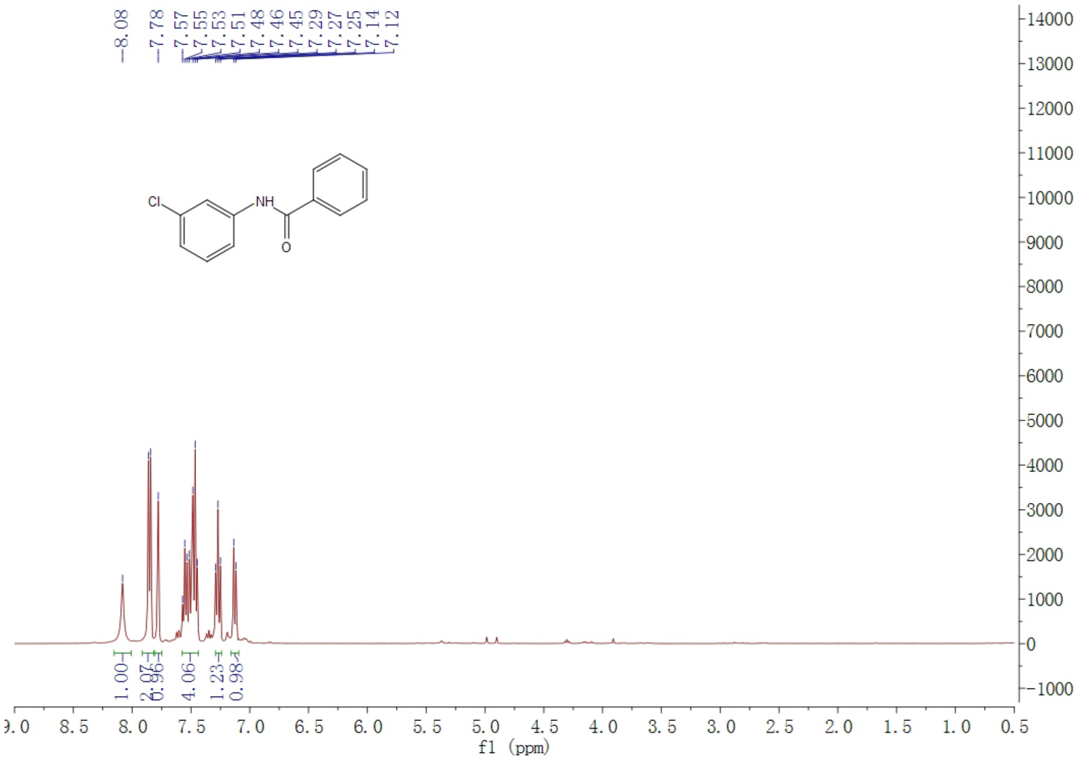

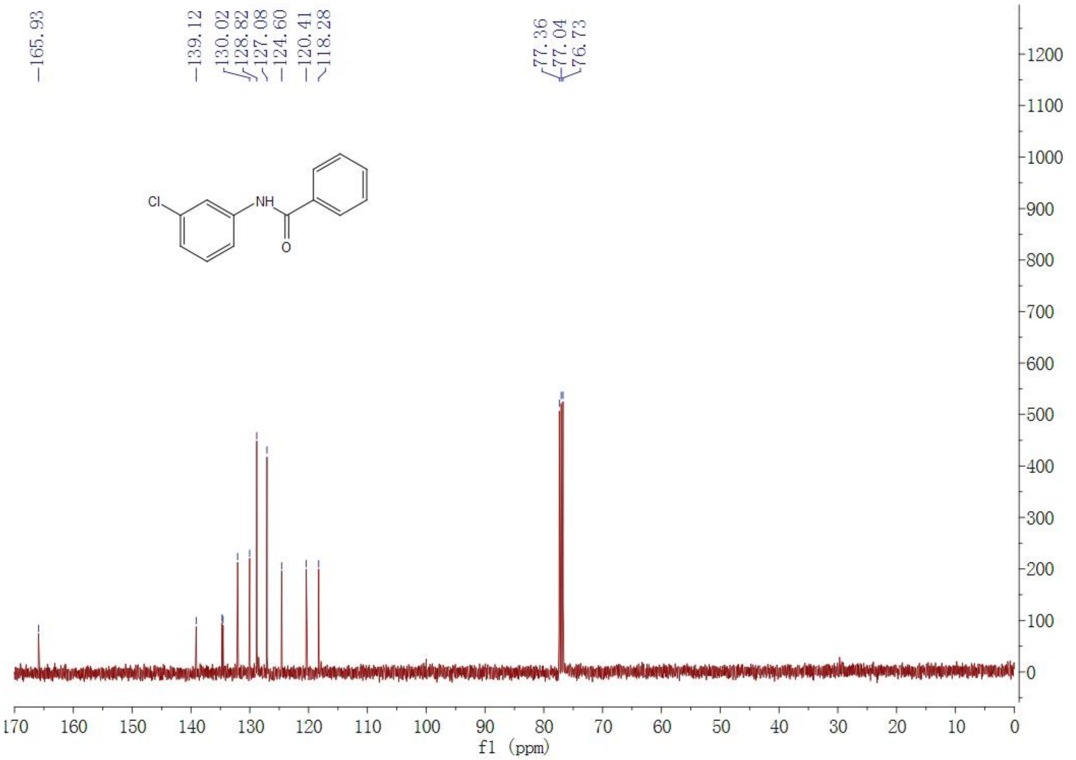

***N*-(4-Chlorophenyl)benzamide (Table 2, entry 5)**

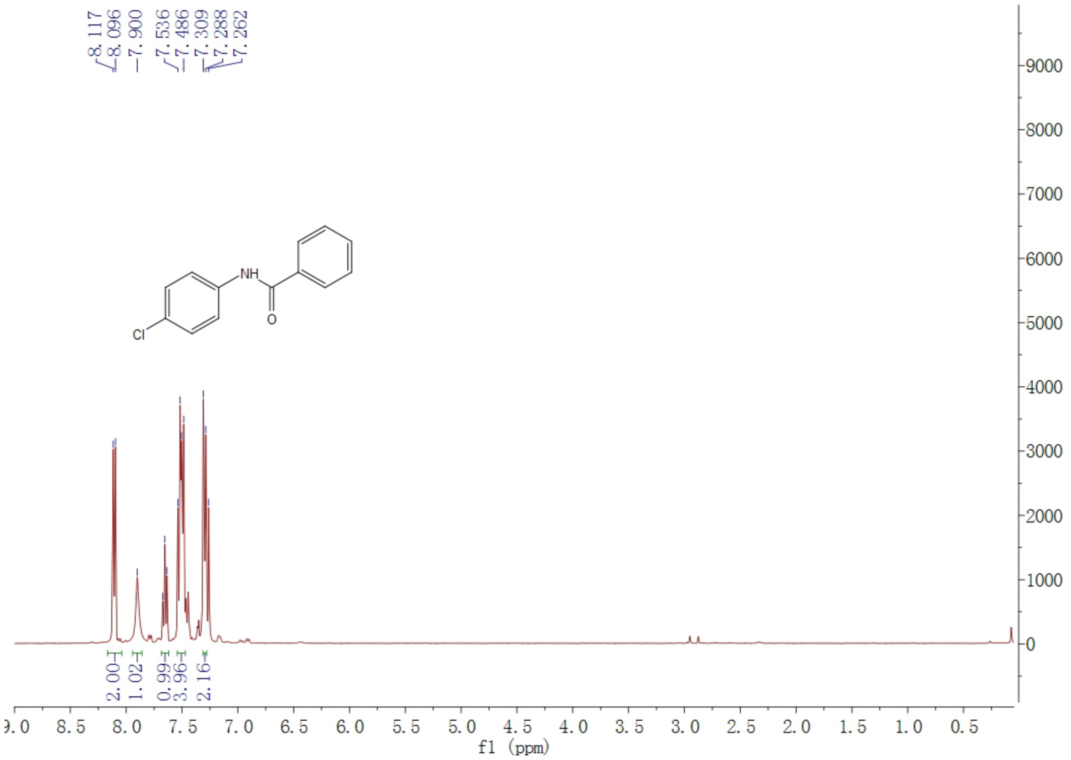

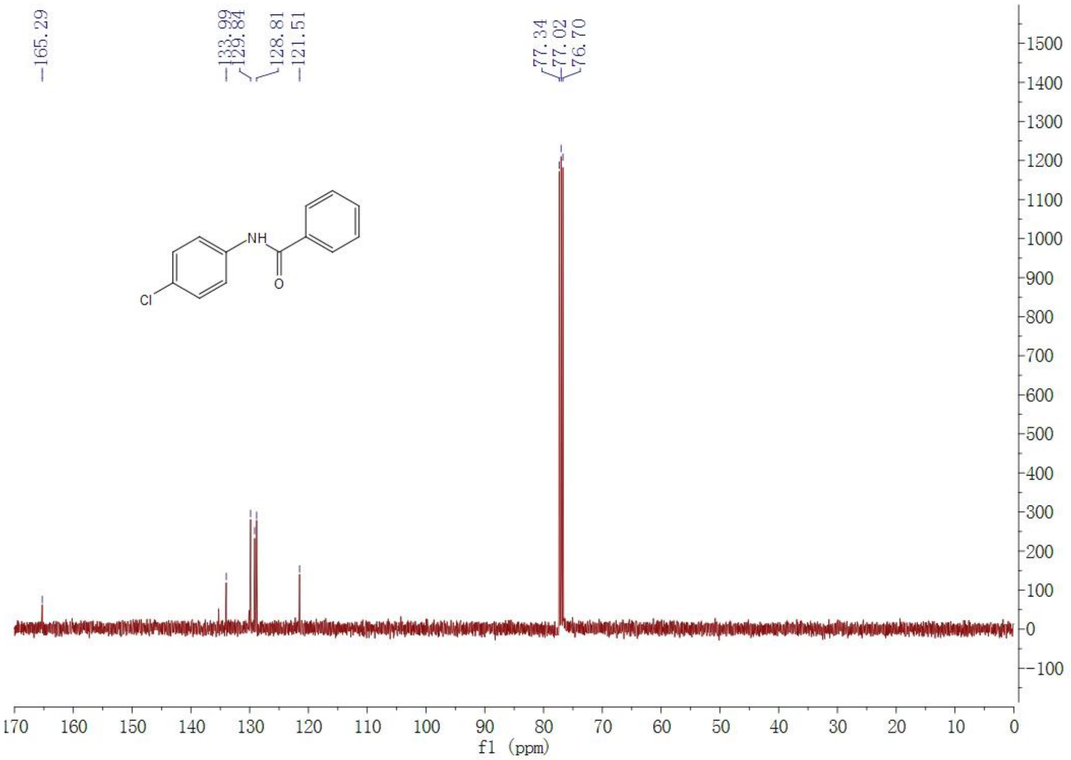

***N*-(4-Methoxyphenyl)benzamide (Table 2, entry 6)**

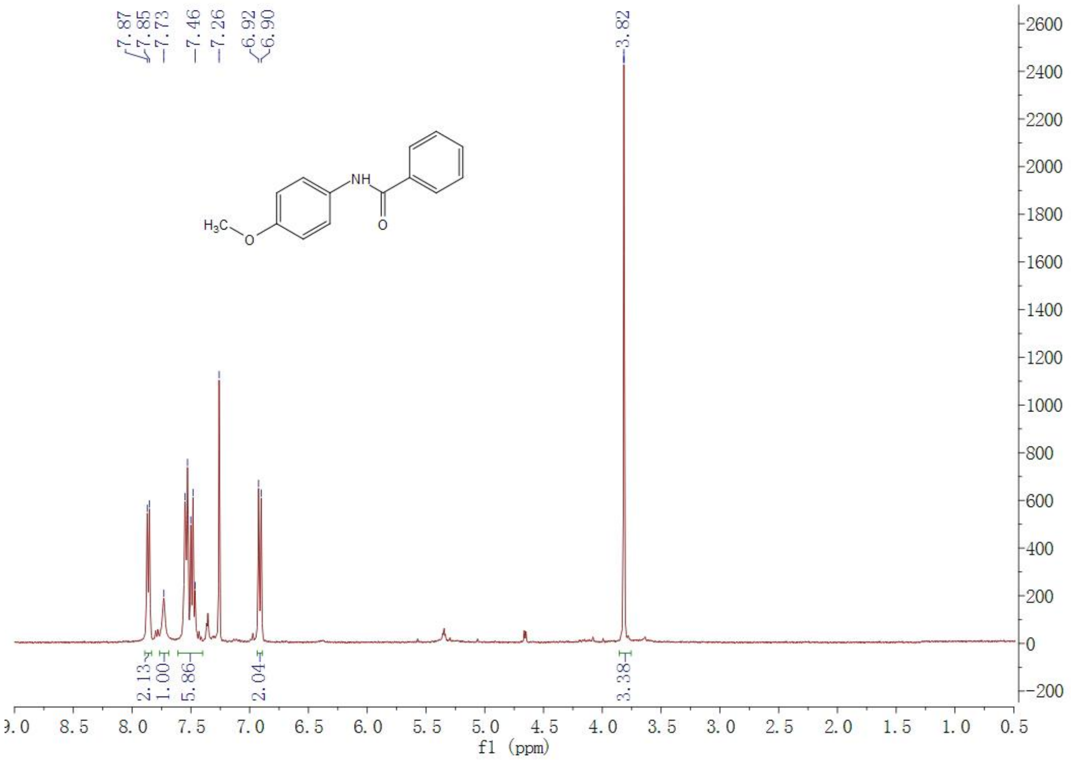

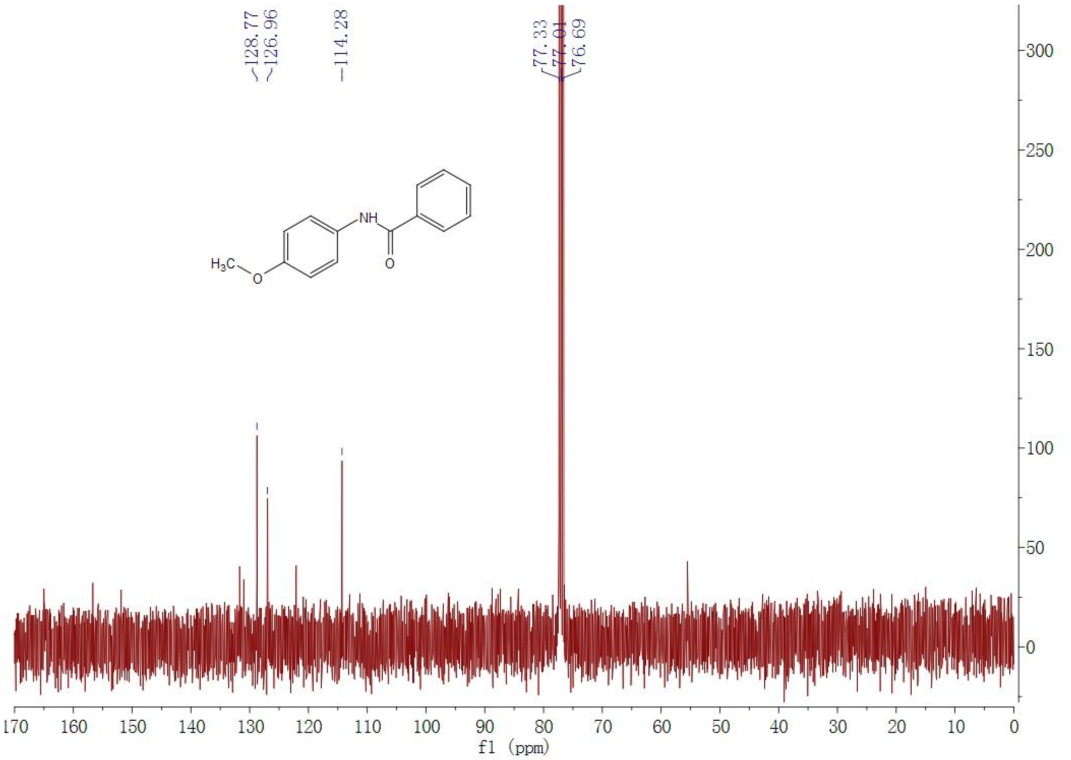

N-(4-Ethoxyphenyl)benzamide (Table 2, entry 7)

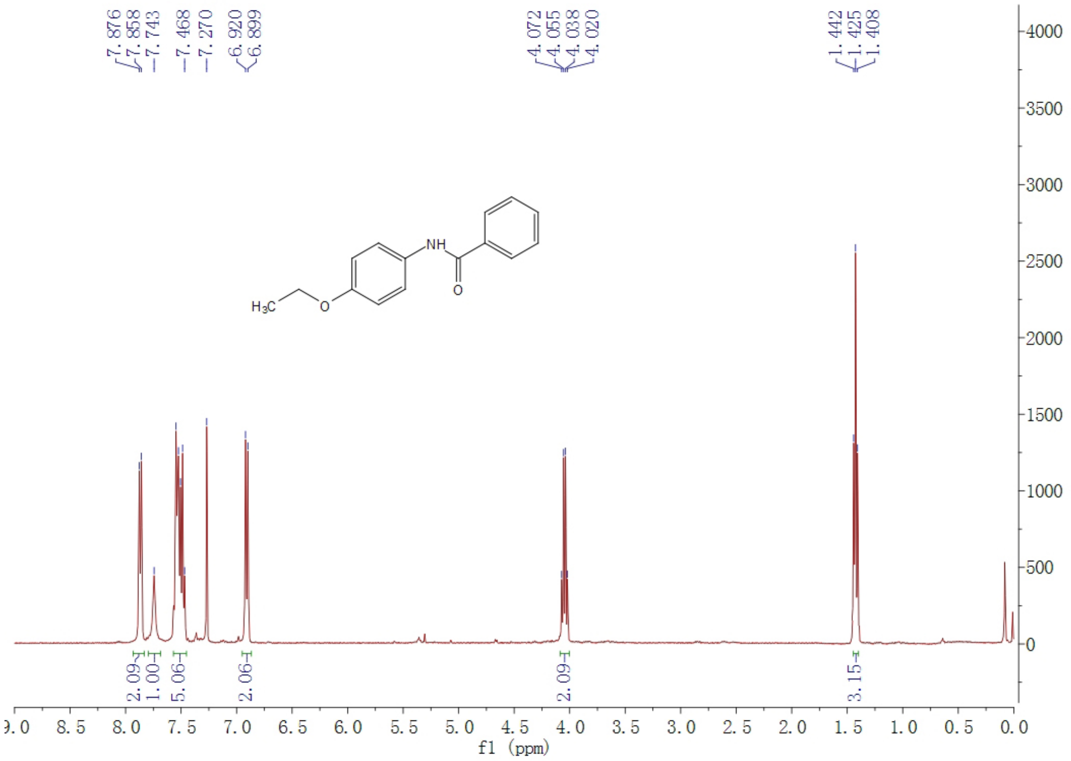

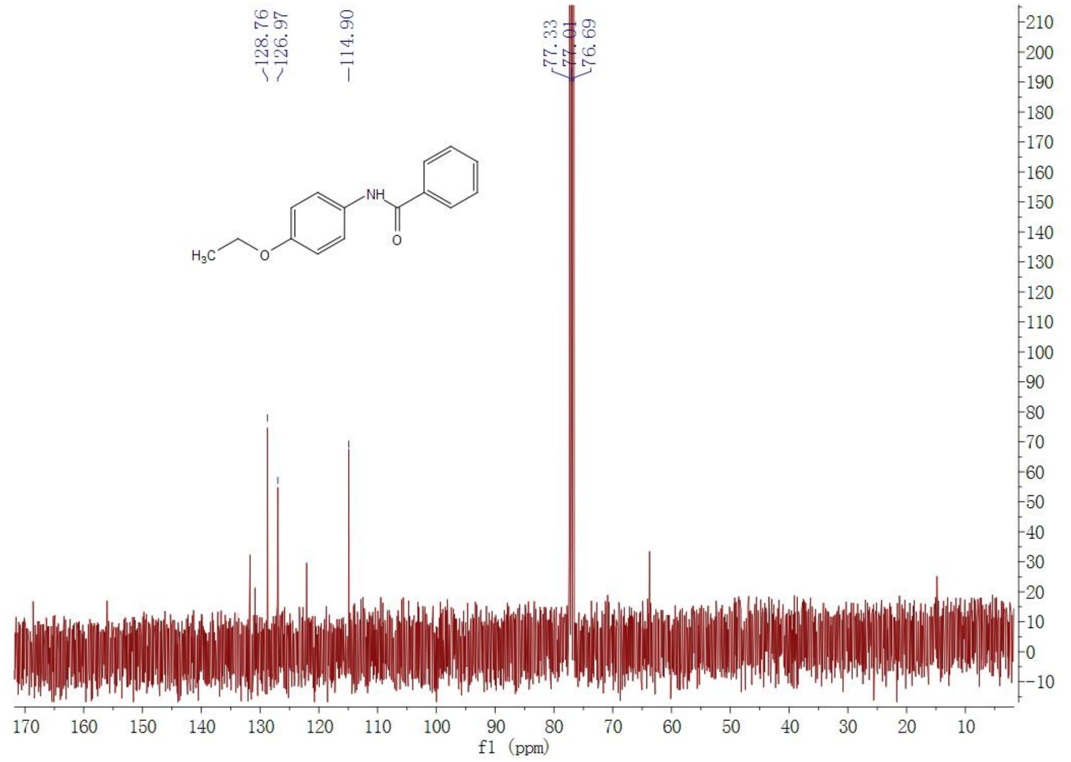

***N*-(4-Cyanophenyl)benzamide (Table 2, entry 8)**

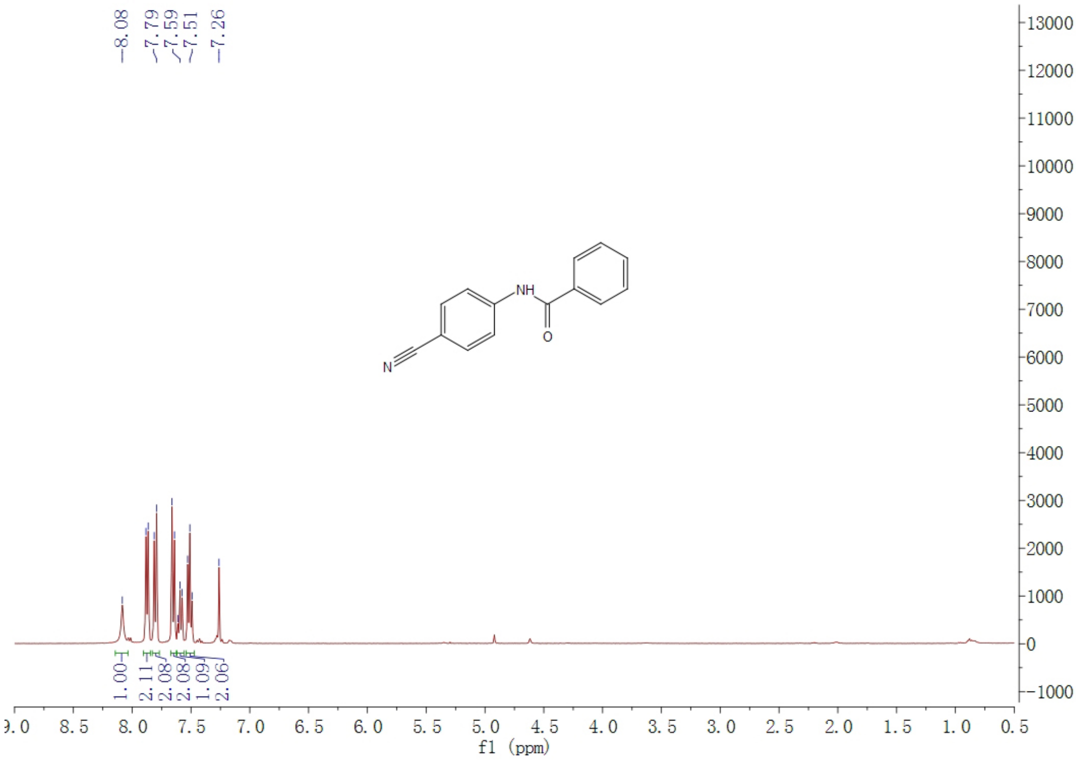

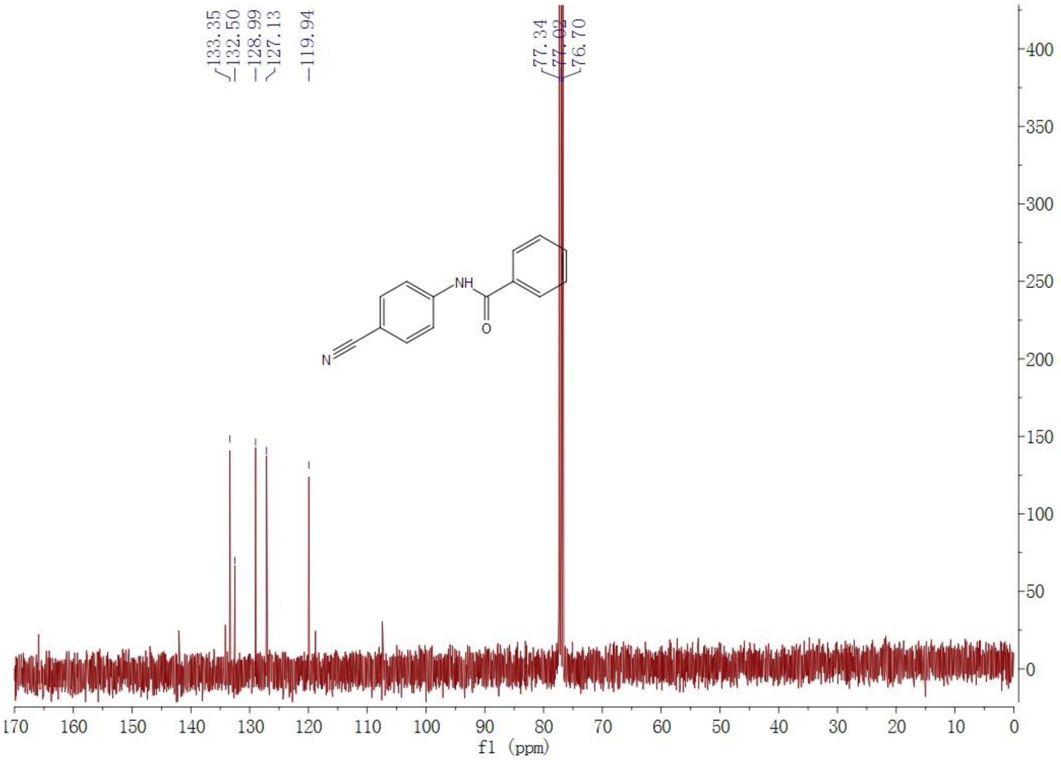

***N*-(*o*-Tolyl)benzamide (Table 2, entry 9)**

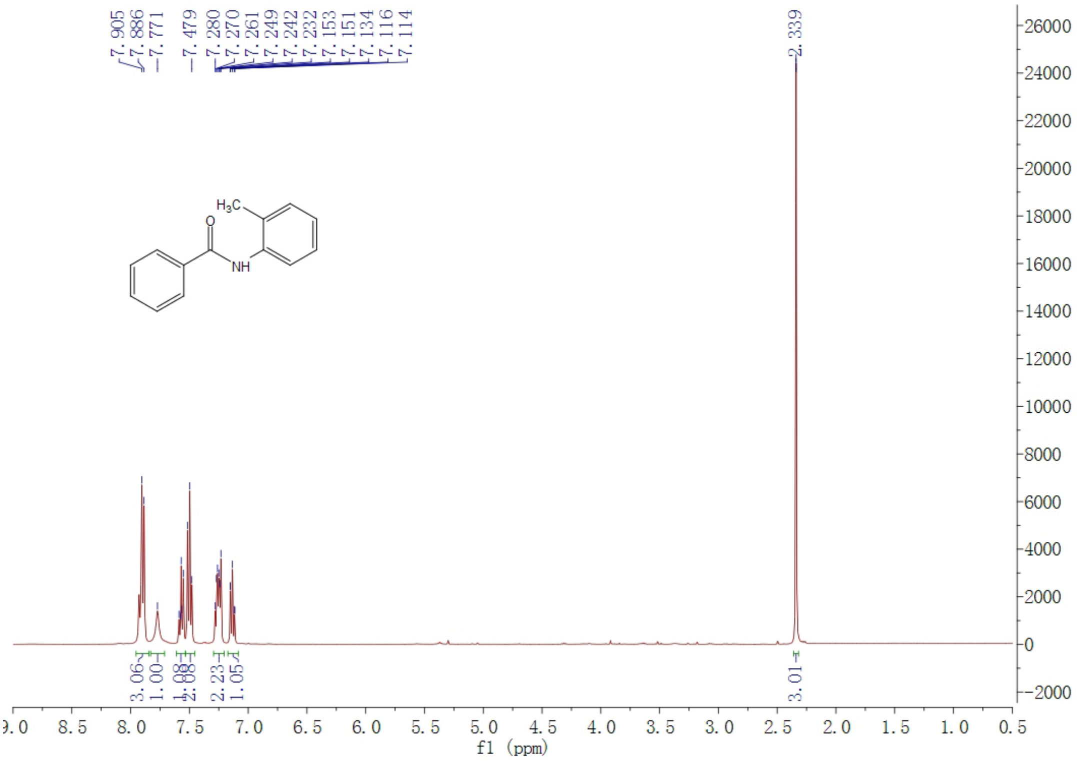

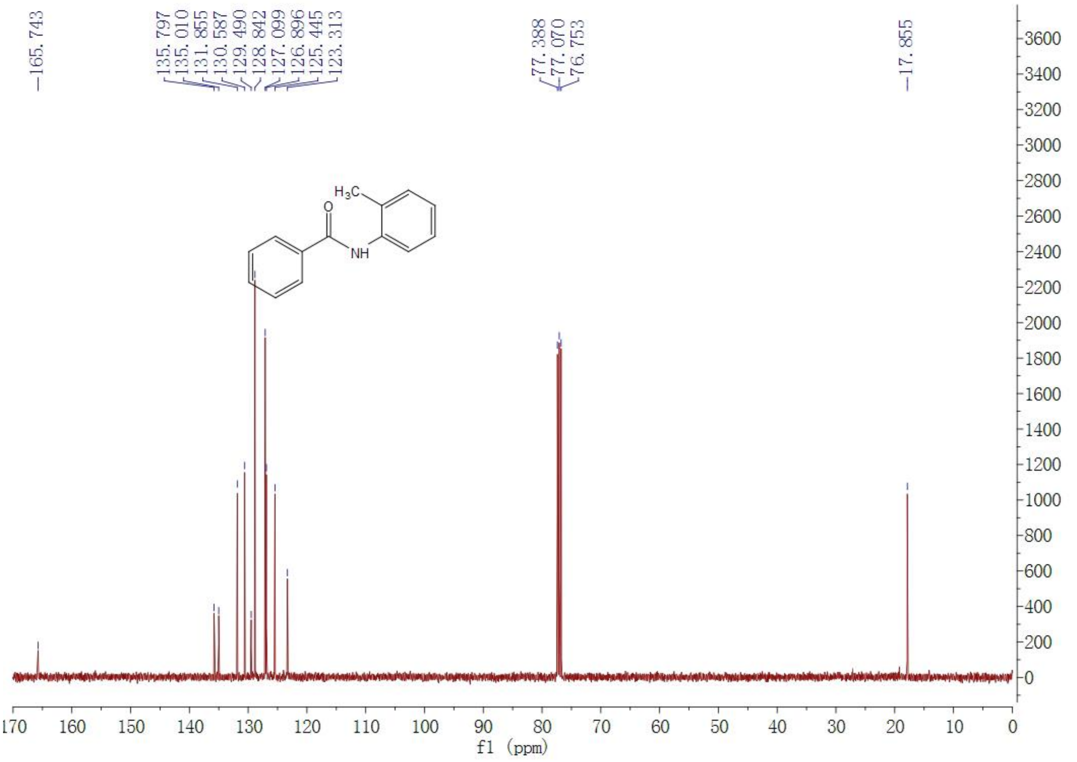

***N*-(*m*-Tolyl)benzamide (Table 2, entry 10)**

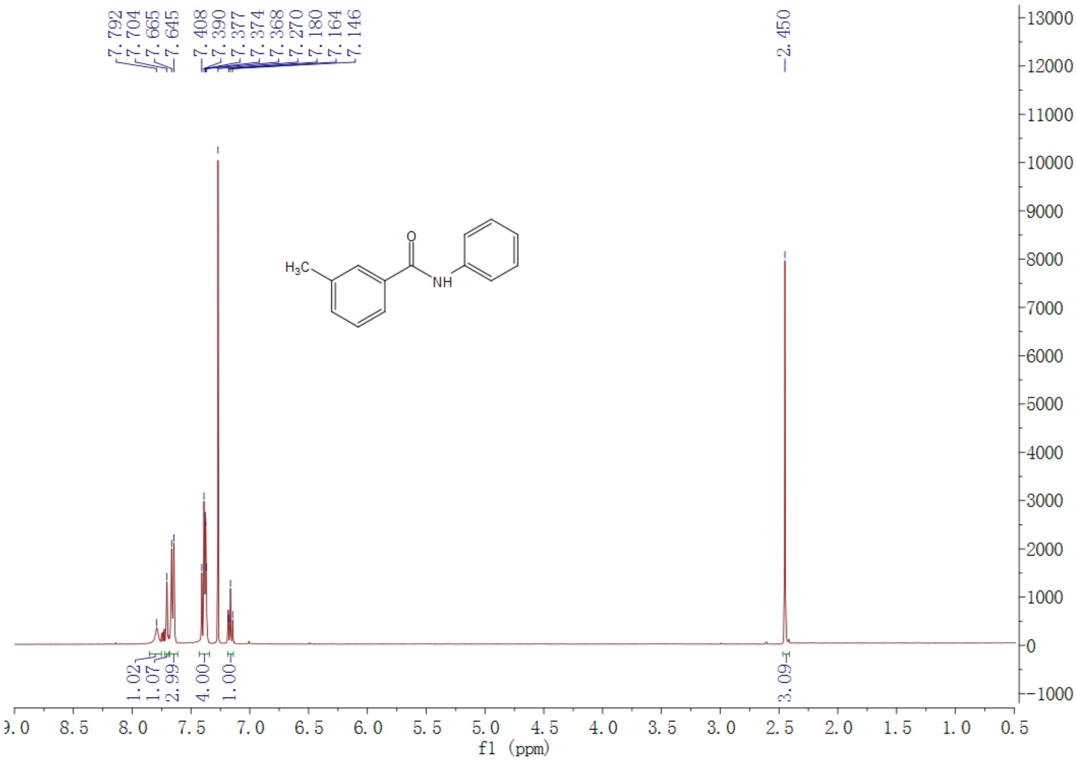

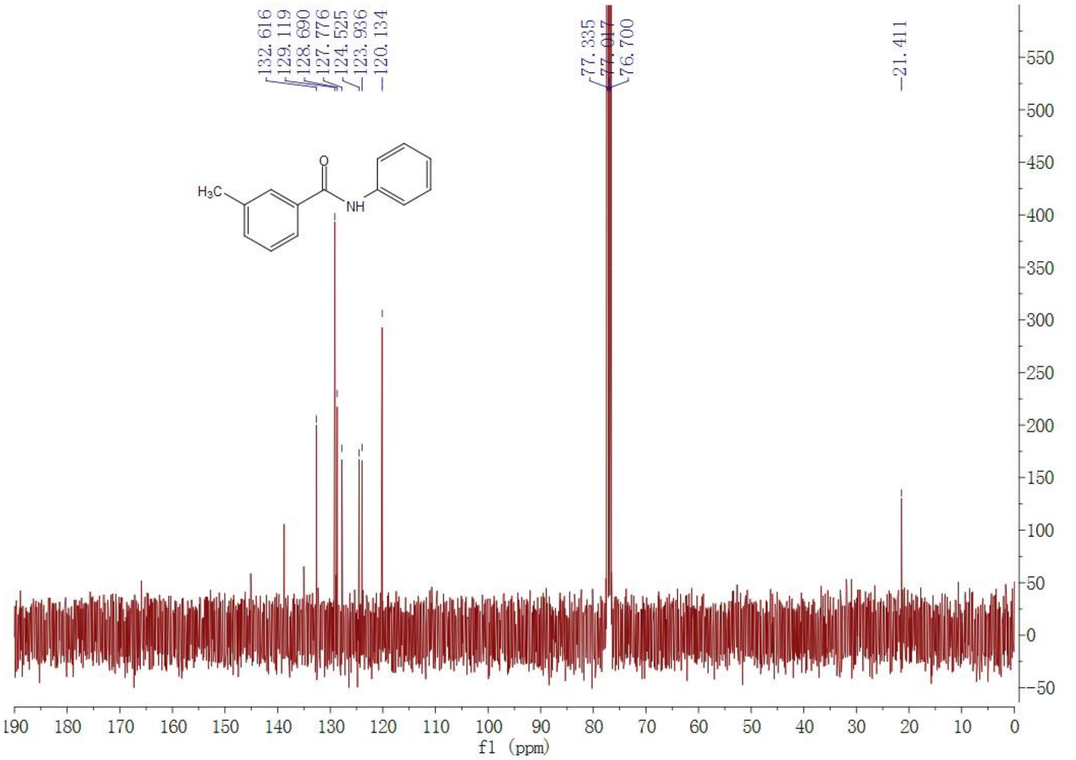

4-Methyl-*N*-phenylbenzamide (Table 2, entry 11)

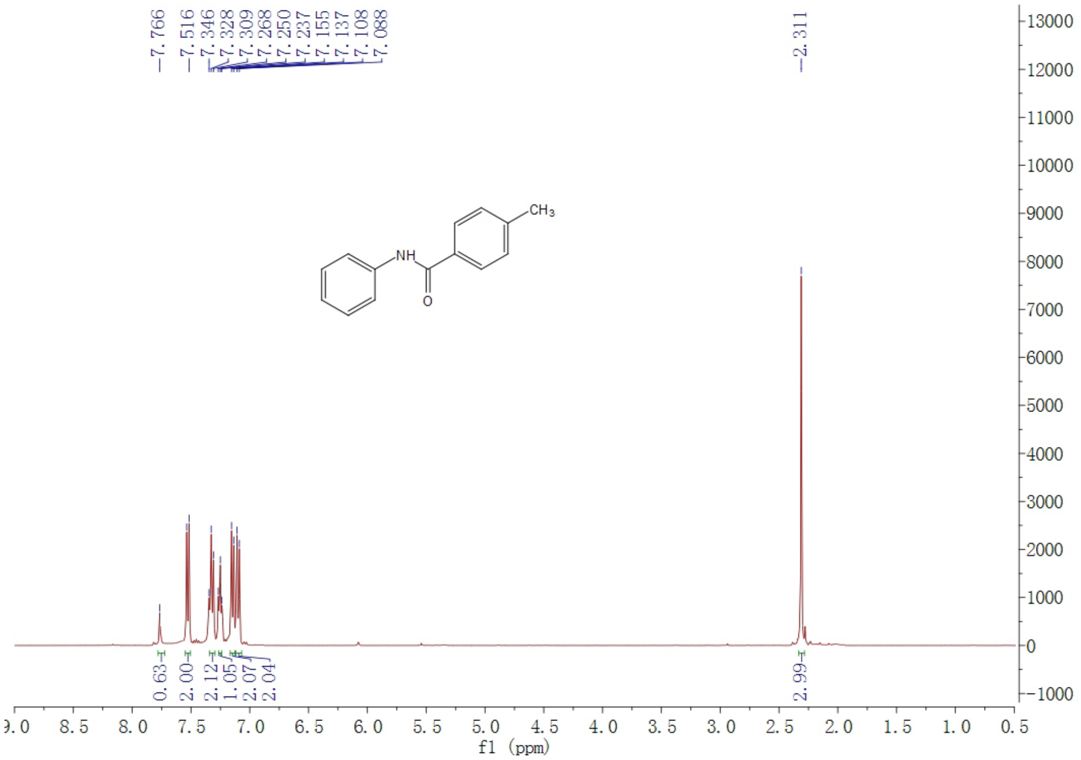

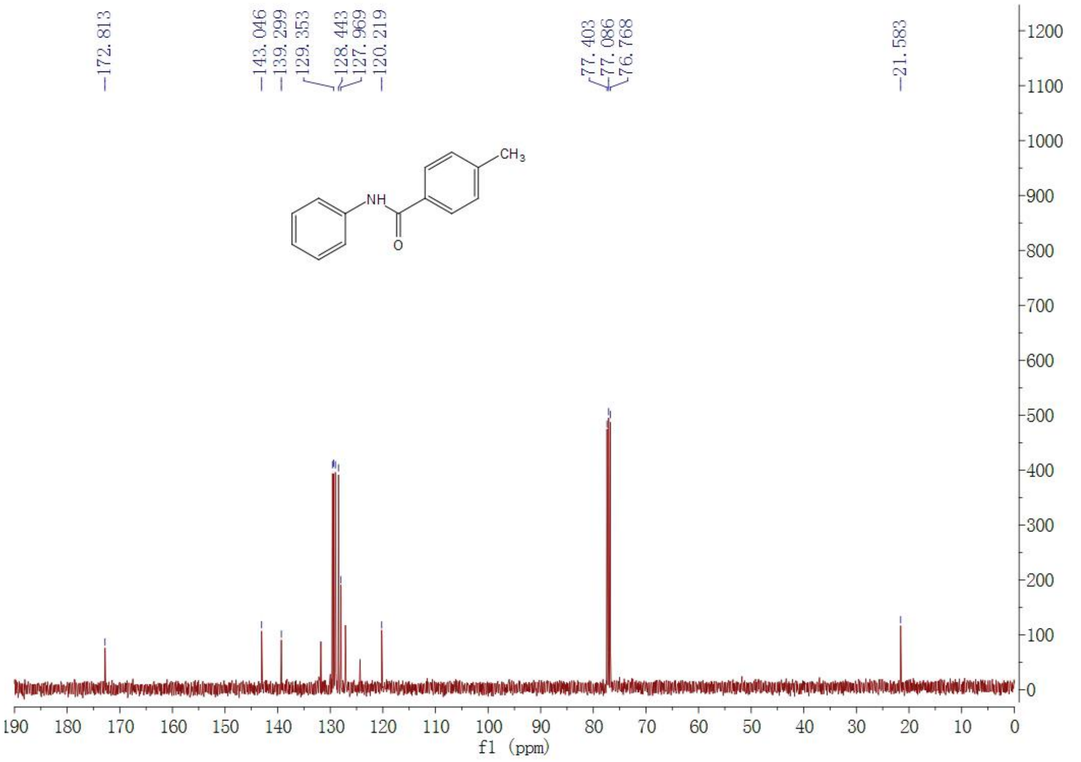

4-Methyl-*N*-(*p*-tolyl)benzamide (Table 2, entry 12)

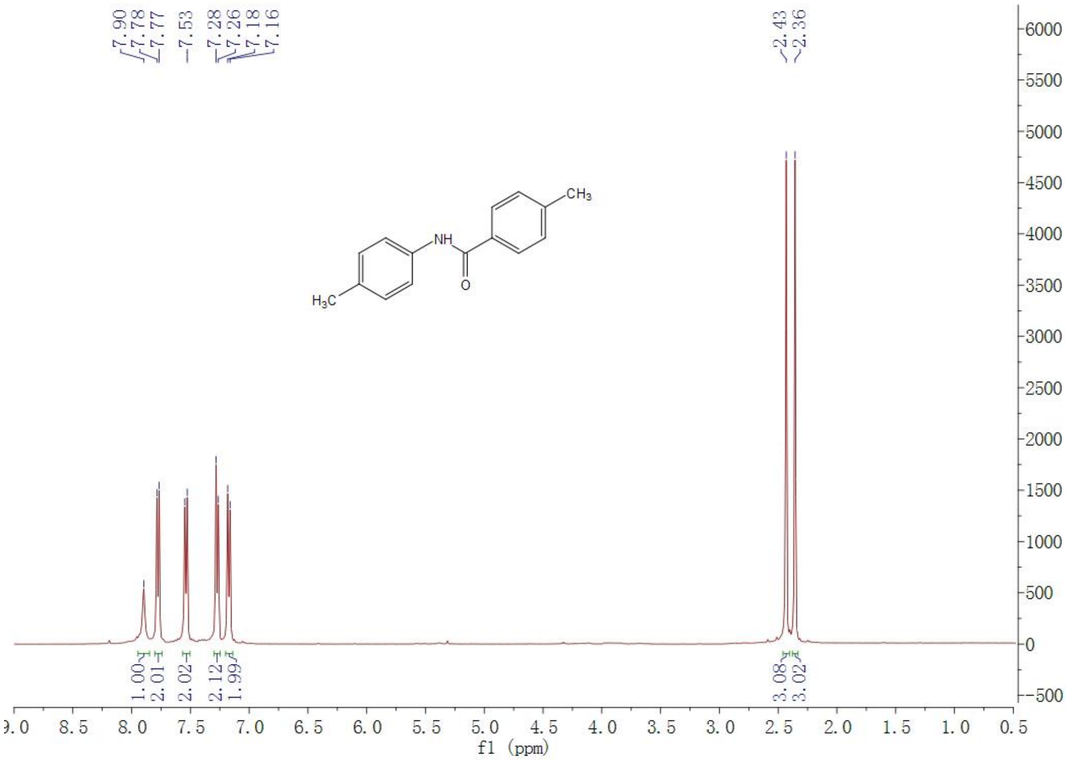

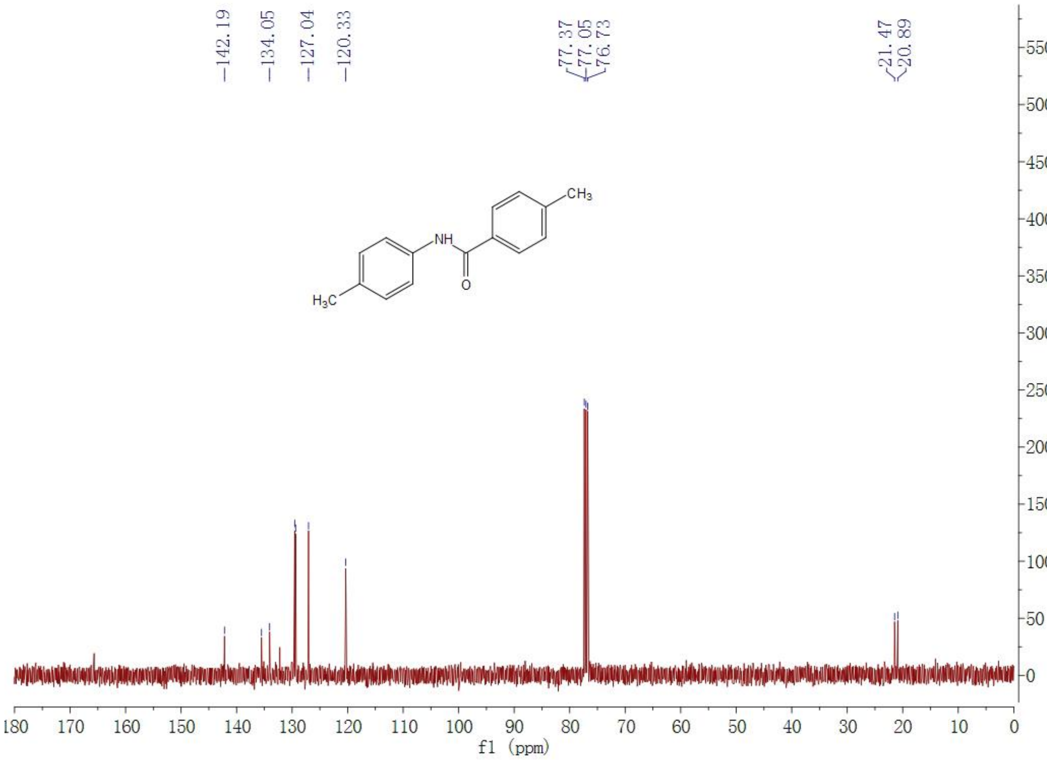

***N*-(2-Chlorophenyl)-4-methylbenzamide (Table 2, entry 13)**

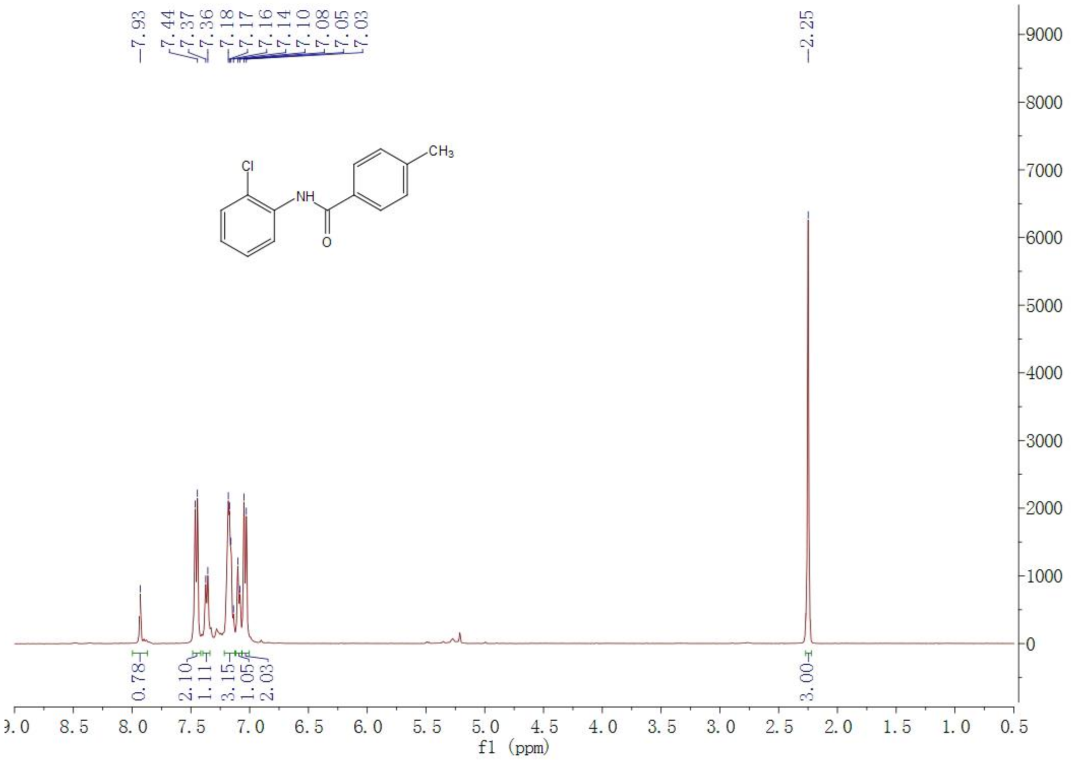

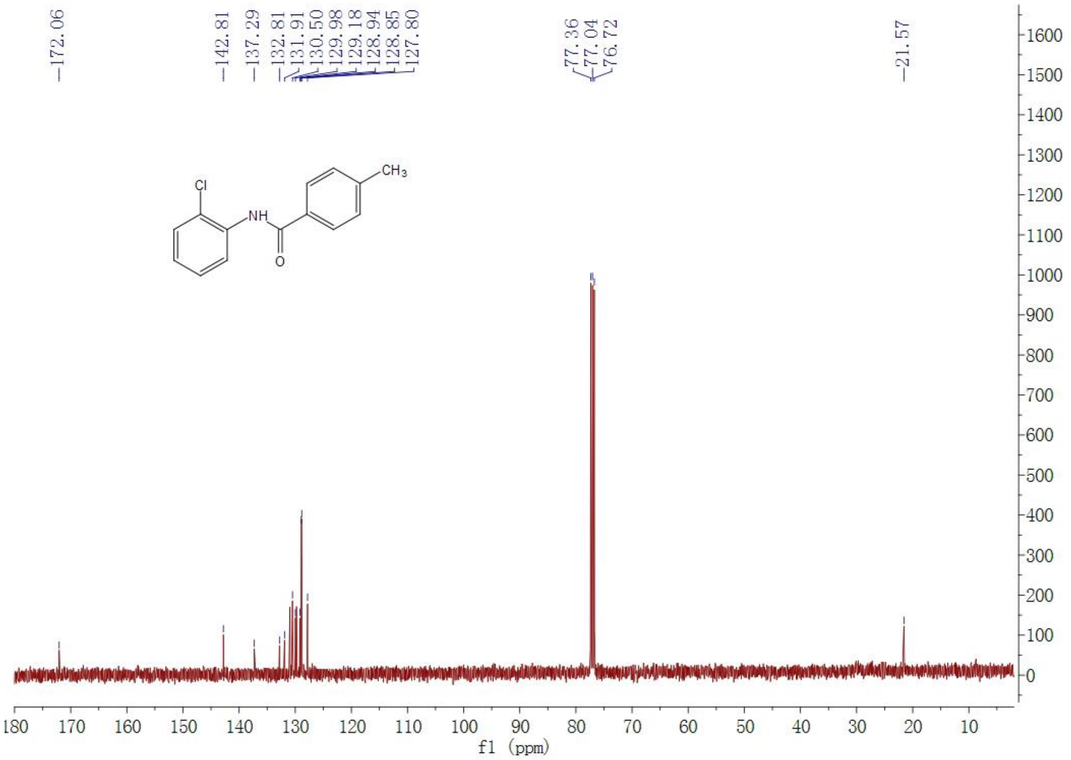

***N*-(3-Chlorophenyl)-4-methylbenzamide (Table 2, entry 14)**

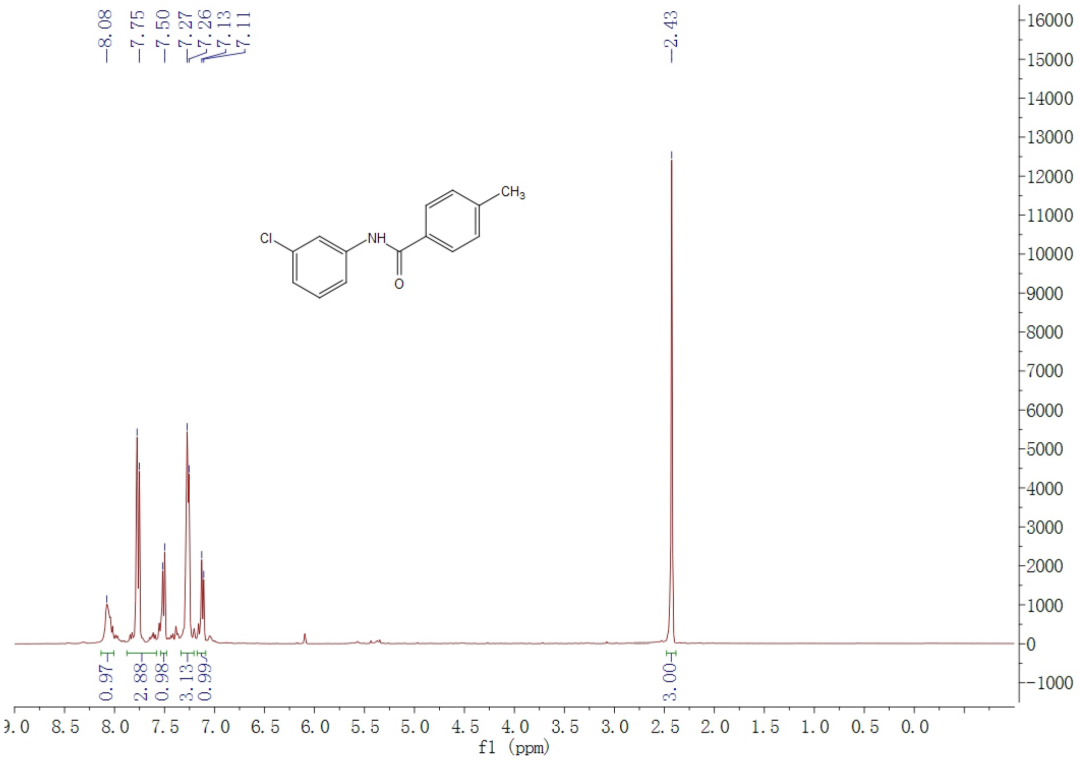

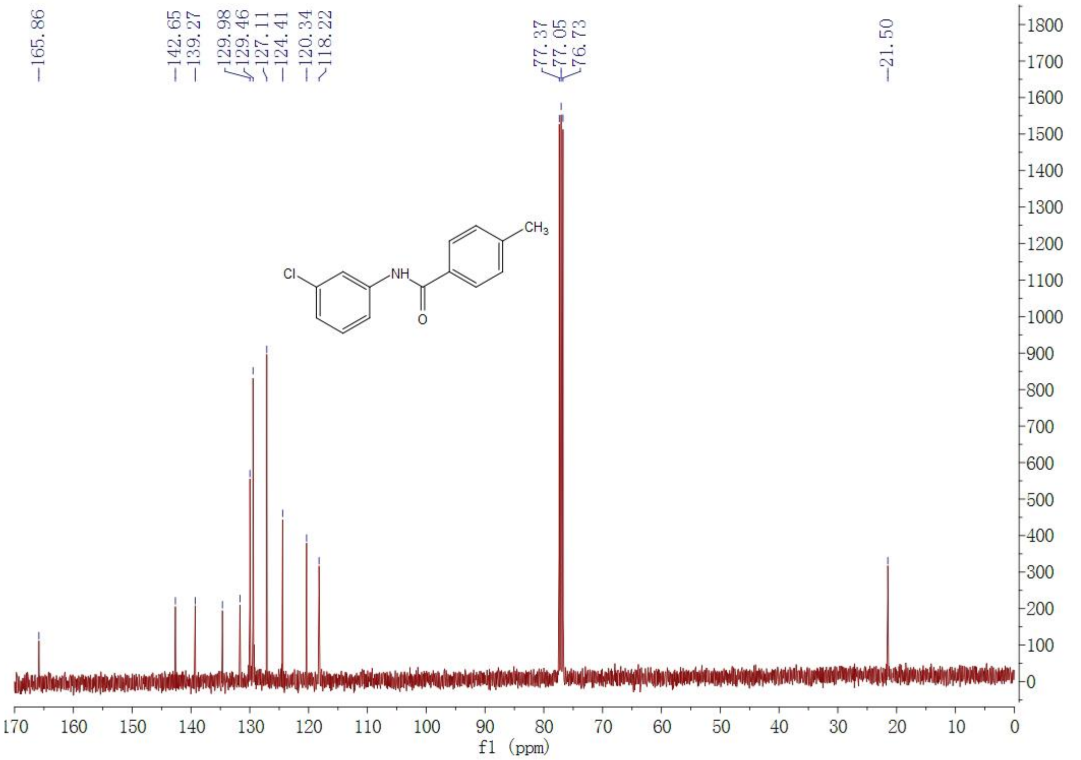

N-(4-Methoxyphenyl)-4-methylbenzamide (Table 2, entry 15)

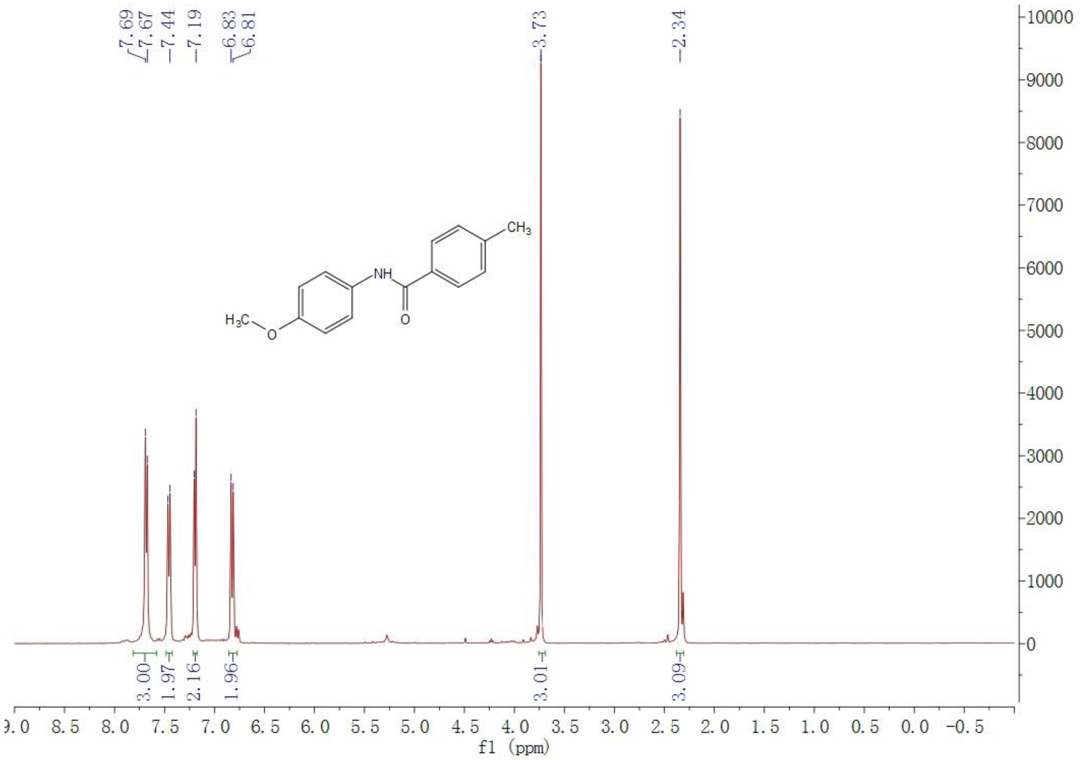

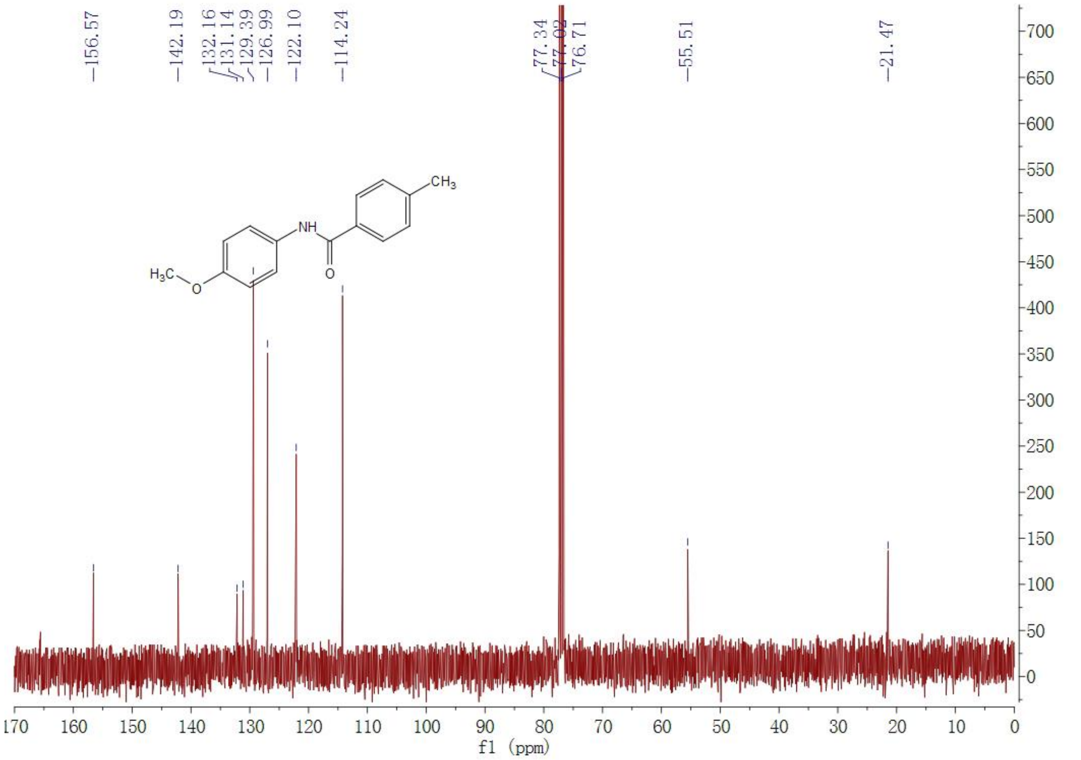

Supplement: File 1 — Full experimental details and copies of NMR spectral data. [file Beilstein_J_Org_Chem-12-2250-s001.pdf]
